# Targeting of short TRPM8 isoforms induces 4TM-TRPM8-dependent apoptosis in prostate cancer cells

**DOI:** 10.18632/oncotarget.8666

**Published:** 2016-04-09

**Authors:** Gabriel Bidaux, Anne-Sophie Borowiec, Charlotte Dubois, Philippe Delcourt, Céline Schulz, Fabien Vanden Abeele, Gilbert Lepage, Emilie Desruelles, Alexandre Bokhobza, Etienne Dewailly, Christian Slomianny, Morad Roudbaraki, Laurent Héliot, Jean-Louis Bonnal, Brigitte Mauroy, Pascal Mariot, Loïc Lemonnier, Natalia Prevarskaya

**Affiliations:** ^1^ Inserm, U-1003, Equipe labellisée par la Ligue Nationale contre le cancer, Villeneuve d'Ascq, France; ^2^ Université des Sciences et Technologies de Lille (USTL), Villeneuve d'Ascq, France; ^3^ Laboratoire de Physique des Lasers, Atomes and Molécules, Equipe Biophotonique Cellulaire Fonctionnelle, Parc Scientifique de la Haute Borne, Villeneuve d'Ascq, France

**Keywords:** TRPM8, ER calcium fluxes, mitochondria, prostate cancer, apoptosis

## Abstract

Since its cloning a decade ago, TRPM8 channel has emerged as a promising prognostic marker and a putative therapeutic target in prostate cancer (PCa). However, recent studies have brought to light the complexity of TRPM8 isoforms in PCa. Consequently, the respective role of each TRPM8 isoform needs to be deciphered prior to considering TRPM8 as an attractive therapeutic target. Full-length (6 transmembrane (TM)-domain) TRPM8 channel is overexpressed in early PCa and repressed in advanced prostate tumors whereas the localization of the truncated, 4TM-TRPM8 channel (4 transmembrane (TM)-domain), in the membranes of endoplasmic reticulum (ER) is independent of the pathogenic status of epithelial cells. In the same line, expression of non-channel cytoplasmic small TRPM8 isoforms (namely sM8) is conserved in cancer cells. In this study, we identify sM8s as putative regulator of PCa cell death. Indeed, suppression of sM8 isoforms was found to induce concomitantly ER stress, oxidative stress, p21 expression and apoptosis in human epithelial prostate cancer cells. We furthermore demonstrate that induction of such mechanisms required the activity of 4TM-TRPM8 channels at the ER-mitochondria junction. Our study thus suggests that targeting sM8 could be an appropriate strategy to fight prostate cancer.

## INTRODUCTION

For 20 years, an increasing number of reports have demonstrated that alterations of calcium (Ca^2+^) homeostasis and/or Ca^2+^ signals interfere with the signaling pathways controlling apoptosis, proliferation, differentiation, secretion and migration [1, 2]. Therefore, with the aim of remodeling the Ca^2+^-transporting proteins network [3], new anti-cancer strategies have emerged, based on genetic invalidation or drug treatment modifying the pathological Ca^2+^ homeostasis in prostate cancer (PCa) cells [4]. Among Ca^2+^ channels, TRPM8 (TRP, Melastatin member 8) is thought to be a putative therapeutic target in PCa [5–8]. Indeed, studies have reported i) an increased expression and activity of TRPM8 channel in the plasmalemma of epithelial cells in intracapsular prostate tumors [9], ii) a strong decrease in TRPM8 expression in androgen-dependent extracapsular PCa and in androgen-dependent metastasis [10], iii) an almost complete suppression of TRPM8 expression in prostates of patients treated preoperatively with anti-androgen therapy [11] due to the absolute requirement of Trpm8 gene for a functional androgen receptor (AR) [8, 12]. In 2007, we reported that a TRPM8 channel truncated isoform was present in the intermediate/transient amplifying normal epithelial prostate cells as well as in PCa and metastasis [9]. Recently, we cloned and characterized this truncated TRPM8 channel isoform in membranes of the endoplasmic reticulum (ER) from human prostate cancer cells and keratinocytes. Interestingly, it exhibits an unconventional structure with 4 transmembrane domains (TMs), and was thus coined 4TM-TRPM8, instead of the 6 TMs characteristic for the TRP channel archetype. We have shown that this 4TM-isoform participates in the regulation of the steady-state [Ca^2+^] in mitochondria and ER [13]. Finally, we cloned and characterized short truncated TRPM8 isoforms, coined short isoforms or sM8. These freely diffusing non-channel isoforms were able to bind to TRPM8 channels in order to differentially modulate their activation by different pathways [14, 15].

Here we have studied the role of these regulatory sM8s subunits of TRPM8 in prostate cancer survival. Using a siRNA-based strategy to decipher their role as non-channel isoforms, we have demonstrated that suppression of sM8 isoforms induced the deregulation of TRPM8 and 4TM-TRPM8 mRNA expression, ER and mitochondrial pathways of oxidative stress, p21 induction and apoptosis. Finally, we have demonstrated that this sM8s-mediated apoptosis in prostate cancer cells required functional 4TM-TRPM8 channels. Altogether, our results suggest that sM8 isoforms participate in resistance against pro-apoptotic signals in prostate cancer cells and consequently that targeting sM8 isoforms rather than the TRPM8 channel itself could be an appropriate and beneficial strategy against extracapsular prostate cancer.

## RESULTS

### Expression of multiple TRPM8 isoforms in prostate cancer cell lines

**Figure 1:**
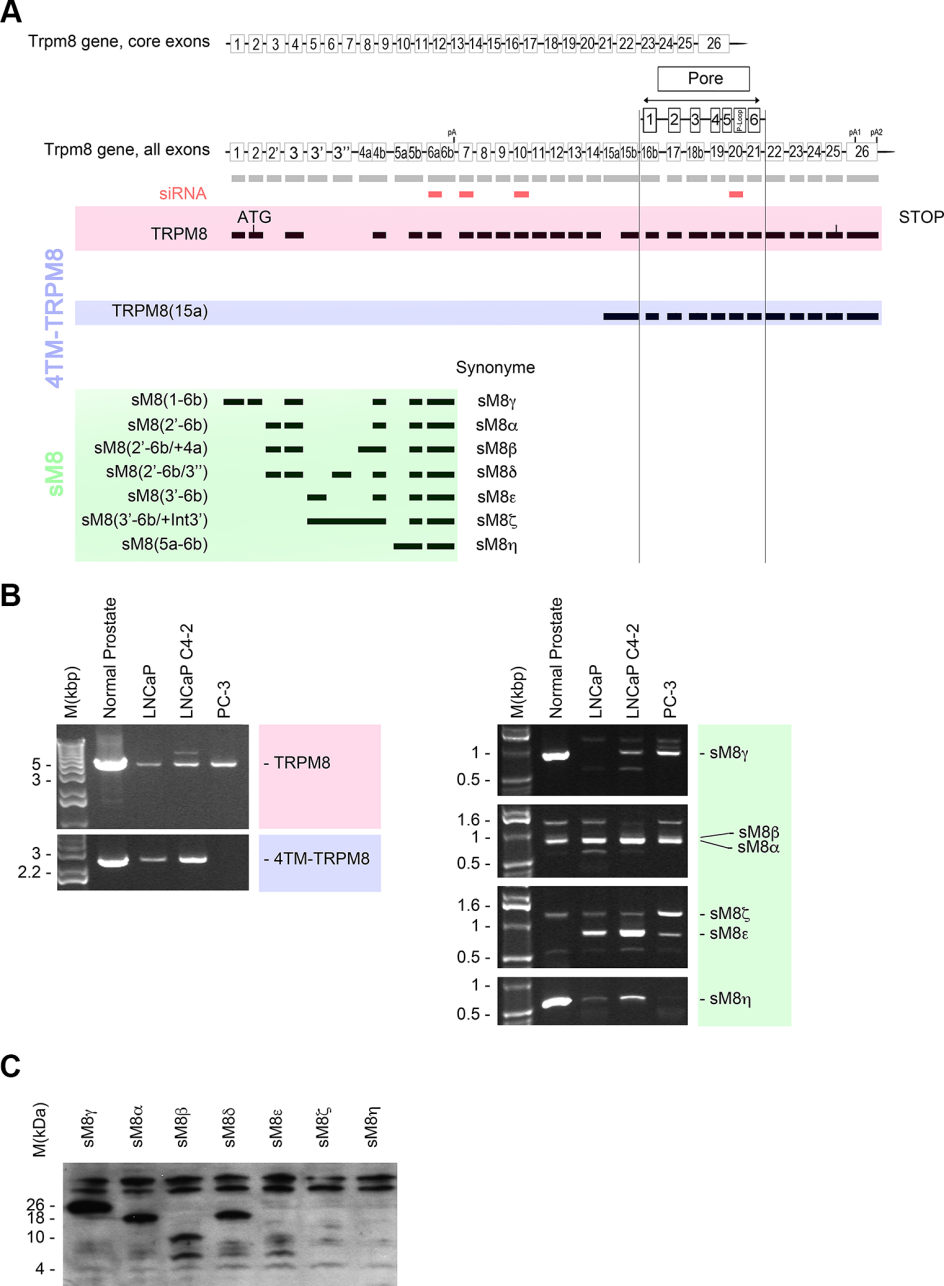
Trpm8 gene encodes 5 alternate TRPM8 mRNA and 2 splice variants in human prostate (**A**) Genomic DNA map (not to the scale) represents the core exons of full-length TRPM8. Alternate exons are labeled with prime or second sign when are forming a cassette exon, and with “a” or “b” when they are forming the 5′ or 3′ supplemental part of a core exon respectively. Transmembrane domains and p-loop of the TRPM8 channel are aligned with their encoding exons. Full-length TRPM8 is showed in the red area (TRPM8) while 4 transmembrane domains TRPM8 isoforms are highlighted in blue (4TM-TRPM8 group) and short TRPM8 isoforms are highlighted in green (sM8 group). siRNA targeting TRPM8 are represented as small red squares aligned with the homologous TRPM8 exons. (**B**) Full-length amplification of TRPM8 cDNAs by PCR reveals the expression of TRPM8 mRNAs in normal prostate tissue, androgen-dependent prostate cancer cell line, LNCaP, androgen-refractory prostate cancer cell line, LNCaP C4-2b, and androgen-independent prostate cancer cell line, PC-3. TRPM8 mRNAs are referenced by their first and last exons, TRPM8 (first exon-last exon). Each PCR reaction was performed with 100 ng of cDNA. (**C**) Western-blot showing the detection of HA-tagged sM8 isoforms in total protein extract of HEK cells. Cells have been transfected with vector encoding sM8 mRNA for 24 h. 75 μg of total protein extract were loaded on each well. Experiments have been reproduced 3 times independently.

In front of the high diversity of TRPM8 mRNA, we rationally coined TRPM8 isoforms based on their structure and deposited them in Genebank as such. However, to facilitate the reading of this study, we also referred to them by shorter Greek letter-based labels (Figure 1A). Accordingly, the TRPM8 isoforms family is composed of i) the TRPM8 channel: the cold and menthol full-length receptor, 130 kDa, referred to as TRPM8 in the text and encoded by the TRPM8(1–26) mRNA (KC692993); ii) a 40 kDa isoform which is a N-terminally truncated cold and menthol activated channel (dubbed eTRPM8 in [13]): referred to as 4TM-TRPM8 in this work, encoded by TRPM8(15a-26) mRNA (KC692994.1). In addition to these channel proteins, we previously cloned and characterized sM8α (sM8(2′-6b); AY532375.1) in the Figure 1A, and sM8β (sM8(2′-6b/+4a); AY532376.1) [15], In the present study, using 5′ RACE-PCR, we have cloned additional sM8 encoding mRNAs, which exhibit alternate start exons (Figure 1A). The five novel sM8 mRNA and accompanying splice variants have been called: sM8γ (sM8(1-6b); KT341002), sM8δ (sM8(2′-6b/+3′′); KT341003) which incorporates the new cassette exon 3′′, sM8ε (sM8(3′-6b); KT341007) the transcription of which starts with the new alternate exon 3′, sM8ζ (sM8(3′-6b/+intron3′; KT341006) characterized by insertion of the intron 3′ and sM8η (sM8(5a-6b); KT341008) whose transcription starts with the new alternate exon 5a (Figure 1A). PCR fingerprinting revealed differences in TRPM8 mRNA expression in human normal prostate as well as in androgen-dependent LNCaP, androgen-refractory LNCaP C4-2b and androgen-independent PC-3 cell lines (Figure 1B). mRNA encoding 4TM-TRPM8 isoform was detected in all samples, except in PC-3 cells. sM8ε was found only in cancer cells while sM8γ and sM8η mRNAs were down-regulated in cancer cell lines compared to normal prostate. sM8 fingerprinting was noticeably divergent between LNCaP and PC-3 cell lines, suggesting that the selection of the first exon could depend on a third party like the androgen receptor. We next assessed which sM8 mRNA could encode proteins by fusing a HA-tag in frame at the C-terminus of the open reading frame. sM8γ was found to code a 26–27 kDa isoform, sM8α and sM8δ coded a 18 kDa isoform, sM8β and sM8ε coded a 9 kDa isoform while sM8ζ and sM8η encoded no protein (Figure 1C). Since sM8s are negative regulatory sub-units of the cold and menthol receptor [15], and because TRPM8 channel has been considered as a target of interest in cancer therapy [16], we assessed whether sM8s knockdown (KD) could affect prostate cancer cell growth.

### sM8 knockdown decreases cell growth of prostate cancer cell lines and induces ER calcium stress

**Figure 2:**
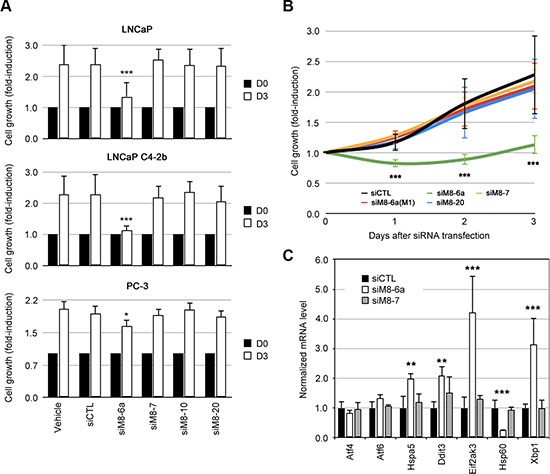
Silencing of sM8 isoforms reduces cell growth of prostate cancer cell lines and triggers stress in endoplasmic reticulum (**A**) Cell growth of LNCaP, LNCaP C4-2b and PC-3 cells was measured with CellTiter 96^®^ AQ_ueous_ Non-Radioactive Cell Proliferation Assay (Promega), after 2 or 3 days of culture. Average values at day 2 and 3 were normalized to values at D0. Experiments were performed three times independently. (**B**) Kinetic of LNCaP C4-2b cell growth following an identical procedure than the one described in A. Cells were transfected with either control siRNA (siLuc) or anti-TRPM8 siRNA (siM8-6a, siM8-7, siM8-20), labels refer to the target TRPM8 exon. A single point mutant siM8-6a showing a limited efficiency, siM8-6a(M1), demonstrates the specificity of the siM8-6a sequence on sM8 KD-mediated inhibition of cell growth. Experiments were performed four times independently. (**C**) Gene expression was estimated with real time PCR after a 3-day siRNA transfection of LNCaP C4-2b cells. Graph plot displays human genes involved in endoplasmic reticulum stress: *ATF4, ATF6, HSPA5, DDIT3 (Chop), EIF2AK3 (PERK), HSP60 and XBP1*. Experiments were performed three times independently. Values are expressed as Mean ± SD.

We postulated that group suppression of TRPM8 isoforms could result in different cellular and molecular phenotypes. We assumed that a subtractive comparison between TRPM8 knocked-down groups should reveal information about the biological function of a specific group of TRPM8 isoforms. For instance, while siM8-6a silenced both sM8s isoform group and full-length TRPM8 channel, siM8-7 silenced only the latter. We therefore inferred that biological variations in sM8-6a knock-down (KD) cells were specific to sM8s suppression. To address this strategy, we developed series of siRNAs targeting TRPM8 exons (and labeled as siM8- exon number) spanned over the TRPM8 gDNA. Silencing efficiency was measured in TRPM8-inducible HEK cells transfected with 50 nM of siRNA for 48 h (Figure S1A). qPCR (Figure S1A) and western-blot (Figure S1B and S1C) experiments validated two siRNAs targeting TRPM8 (siM8-7 and siM8-10), three siRNA targeting the sM8s in addition to TRPM8 (siM8-4b, siM8-6a and siM8-6a.2) and one siRNAs targeting 4TM-TRPM8 in addition to TRPM8 (siM8-20). The specificity of siRNA is usually guaranteed by the use of low siRNA concentrations (below 100 nM), and by comparing their biological effects with the ones induced by control siRNAs. This latter is usually assumed to be a RNA sequence which does not match with genome sequences of the host organism. However, this does not completely guaranty the absence of off-target effects. A further control could be achieved by the mean of degenerated sequences, which only diverge from the original siRNA by one or two base substitutions. We therefore designed two siM8-6a mutants and showed that they failed to significantly silence sM8 mRNA (Figure S1D). Two studies have reported that the inhibition or suppression of TRPM8 reduces both cell survival [12] and proliferation of LNCaP cells [17]. On the contrary, in all prostate cell lines that we have tested here, the suppression of either TRPM8 alone (with siM8-7 or siM8-10) or TRPM8 and 4TM-TRPM8 isoform together (with siM8-20) did not modify cell growth (Figure 2A). On the contrary, the concomitant suppression of both sM8 group and TRPM8 with siM8-6a resulted in a strong reduction of cell growth in LNCaP and LNCaP C4-2b (Figure 2A). Because the suppression of TRPM8 alone did not modify cell growth, one might conclude that the decrease in prostate cell growth induced with siM8-6a originates from the suppression of the sM8 group. Cell growth kinetic studies showed a cytostatic effect of sM8 KD in LNCaP C4-2b (Figure 2B) and LNCaP cells (Figure S2A) cells. Mutant siM8-6a(M1) did not modify growth of LNCaP C4-2b (Figure 2B) or LNCaP cells (Figure S2A). In PC-3 cell line, which did not express 4TM-TRPM8, sM8-6a and siM8-6a(M1) induced a low and similar decrease in cell survival (Figure S2B). These results, altogether, demonstrate high sM8-KD specificity of siM8-6a in LNCaP C4-2 cells while a low sM8-unspecific effect could be observed in PC-3 cells. We next injected LNCaP C4-2b cells subcutaneously in male nude mice and reported their growth rate. Surprisingly, no significant change in tumor growth rate was observed with either siCTL or siM8-6a (Table 1).

**Table 1:** Mean rate of C4-2 tumor volume (mm^3^/day) in mice

wt C4-2	siCTL	siM8-6a
Mean	37.31	32.53
SD	6.80	8.88
*t*-test		0.2372
Sample	9	9
		
sM8 α C4-2	siCTL	siM8-6a
Mean	27.48	12.25
SD	14.40	10.69
Unpaired *t*-test (two tails)		0.0152
Sample	10	10

Microscopic observation of LNCaP C4-2b cells revealed that siM8-6a treatments produced cell unhealthiness (Figure S2C) characterized by retraction of cell extensions, loss of contrast, and detachment from the underlying matrix, while siM8-7 (TRPM8 KD cells) did not alter cell morphology. Mutant siM8-6a(M1) also led to cell alterations, though less pronounced than those induced by siM8-6a. Faced with this evidence and given the localization of TRPM8 and 4TM-TRPM8 at the ER membranes, we checked for ER stress induction. We detected by qPCR a significant induction of ER stress markers Hspa5, Ddit3 (CHOP), Eif2ak3 (PERK), and spliced Xpb1 (Figure 2C), and an increased expression of GRP78 protein (Figure S3A) as well. Although expression of mitochondrial stress markers was unchanged (Figure S3B), a strong reduction of Hsp60 was detected - reporting oxidative stress in mitochondria (Figure 2C). Among the three ER stress sensors: IRE1α (inositol-requiring protein-1α), PERK (protein kinase RNA (PKR)-like ER kinase), and ATF6 (activating transcription factor 6), PERK pathway has been implicated in survival/apoptosis response to oxidative stress (for review, see [18, 19]). Since both ER stress and oxidative stress are known inducer of cell cycle arrest and apoptosis [18, 20–22], we wondered whether the decrease in cell growth was related to cell cycle arrest or to cell death.

### sM8 knockdown induces PERK-mediated apoptosis and p21 expression in quiescent LNCaP C4-2b cells

**Figure 3:**
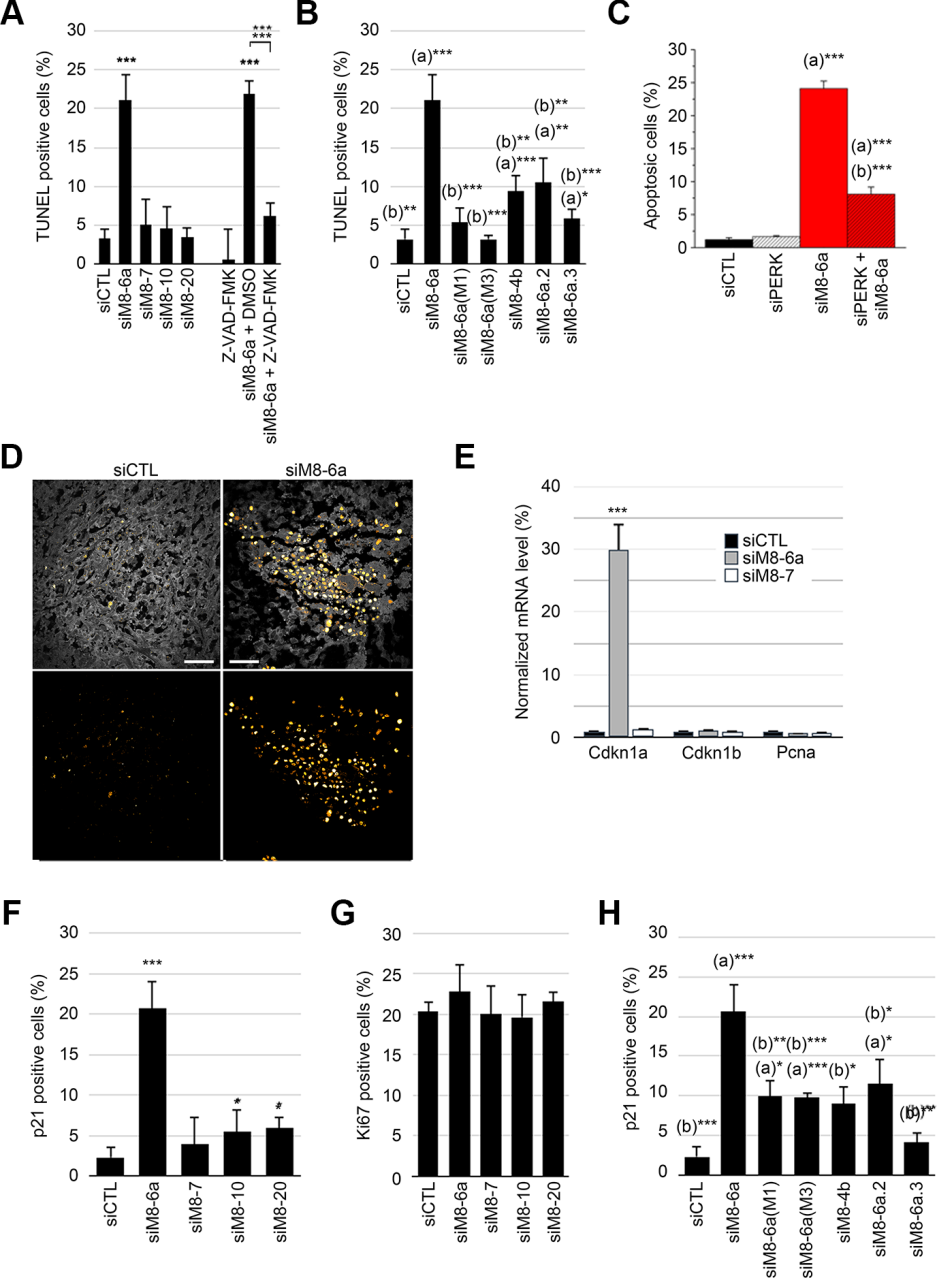
Silencing of sM8 isoforms induces apoptosis and increases p21 positive cell population of prostate cancer cells (**A**) Terminal deoxynucleotidyl transferase dUTP nick end labeling (*TUNEL*) reaction was performed on LNCaP C4-2b cells with nucleotide coupled to tetramethyl rhodamine (TMR) before cell counting was achieved by flow cytometry. Graph plot represents the proportion of apoptotic cells. This reveals an induction of apoptosis after a 3-day transfection with siRNA targeting TRPM8 exon 6a. No significant variation of apoptosis is detected with other anti-TRPM8 siRNA (siM8-7, siM8-10, siM8-20) compared to the control siRNA (siLuc). This induction of apoptosis is strongly reduced in cells treated with the general inhibitor of caspases: Z-VAD-FMK. (**B**) Percentage of apoptotic cells is increased by transfection of other siRNA targeting other exons of sM8s other than exon 6a, while mutants of the siM8-6a siRNA failed to trigger apoptosis. Statistical test with siCTL: (a), and with siM8-6a: (b). (**C**) The concomitant suppression of PERK (siPERK) and sM8s (siM8-6a) decrease sM8 KD-induced apoptosis by 66% in LNCaP C4-2b cells. Cells were stained with Hoechst and the count of apoptotic nuclei was normalized by the count of total nuclei. Statistical test with siCTL: (a), and with siPERK: (b). (**D**) TUNEL reports the detection of apoptotic cells in subcutaneous LNCaP C4-2b tumors. Left panels show tumors in mice injected with CTL siRNA and right panels show low growth rate tumors developed in siM8-6a-injected mice. Top panels are merges of autofluorescence image (excited at 488 nm, gray scale image) and TUNEL images (excited at 633nm), while bottom panels only show TUNEL signal (Orange Hot LUT). Scale bars: 30 μm. (**E**) Gene expression was estimated with real time PCR after a 3-day siRNA transfection of LNCaP C4-2b cells. Panel shows genes coding for cell cycle inhibitor p21 and p27^kip^ (respectively *CDKN1A* and *CDKN1B*), and the *PCNA* gene coding for the proliferating cell nuclear antigen, which is expressed during cell cycle. (**F**) and (**G**), show an increase in the proportion of p21 and Ki67 positive cells, respectively, after transfection of cells with siRNA targeting different groups of TRPM8 isoforms. Cells were sorted by flow cytometry after immunolabeling of p21 (Alexa647) and Ki67 (Alexa488) proteins. (**H**) The specificity of sM8 KD-induced p21 expression was assessed by the use of alternate siRNA, as described in B. The proportion of p21 immunolabeled cells was counted as explained in F. Experiments were performed three times independently. Values are expressed as Mean ± SD.

Apoptotic cells labeled by terminal deoxynucleotidyl transferase dUTP nick end labeling (*TUNEL*) have been sorted out by flow cytometry analysis. As reported in Figure 3A, siM8-6a treatment increased apoptotic cell population to 21.12 ± 3.39%, a Figure reduced to 6.12 ± 1.79% in the presence of the broad-spectrum caspase inhibitor Z-VAD.FMK. siM8-6a mutants (M1 and M3) failed to induce apoptosis in LNCaP C4-2b cells (Figure 3B). Supplemental siRNAs targeting both sM8 isoforms and full-length TRPM8 channel (siM8-4b, siM8-6a.2 and siM8-6a.3) significantly increased apoptosis above the control level, although less efficiently than siM8-6a (Figure 3B). Finally, we assessed whether the oxidative-mediated ER-stress pathway could be involved. We show (Figures 3C and S3C) that siM8-6a-induced apoptosis could be strongly reduced by a concomitant suppression of PERK. This demonstrates the requirement of the PERK pathway in the triggering of ER stress-associated sM8 KD-dependent apoptosis of LNCaP C4-2b cells. Although siM8-6a treatment failed to reduce growth of *in situ* C4-2b tumors, TUNEL assay performed on tumor slices revealed a strong induction of apoptosis in siM8-6a injected mice compared to CTL mice (Figure 3D). This suggests that sM8s KD efficiently induced apoptosis in tumors, but that its overall effect on tumor growth was counterbalanced by unidentified mechanisms, specifically in an *in vivo* environment.

Though apoptosis could explain the cytostatic effect reported in Figure 2B, we next checked whether siM8 KD induced a parallel decrease in cell proliferation. We focused on p21, a protein restricting cell cycle at both G1/S and G2/M transition [23, 24] and participating in apoptosis induction [25–27] and on Ki67, a pro-proliferative protein expressed from G1/S checkpoint until the exit of mitosis [28]. As shown in the Figure 3E, siM8-6a treatment induced a robust increase in *Cdkn1a* expression, the p21-coding gene. Using flow cytometry (FACS), we estimated the proportions of cell population expressing both the anti-proliferative p21 protein and the pro-proliferative Ki67 protein. The proportion in p21 positive cells increased to 20.63 ± 3.53% after sM8 KD (Figure 3F and 3G) but the proportion of Ki67 positive cells was stable. By contrast, TRPM8 KD or 4TM-TRPM8 KD induced p21 expression in 5.46 ± 1.51% and 6.02 ± 1.29% of cells, respectively. This p21 induction was significantly lowered with siM8-6a mutants (10.0 ± 4.15% (M1) and 9.87 ± 0.84% (M3)), (Figure 3H). Besides, siM8-4b and siM8-6a.2 also increased p21 expression, even though less efficiently than siM8-6a. The dual distribution of p21 and Ki67 labeled cells revealed that sM8 KD mediated a strong increase of p21 in Ki67 negative cells (Figure S4A). In order to confirm this paradoxical result, we performed a cell cycle analysis by FACS. Cell cycle analysis was carried out on LNCaP C4-2b cells labeled with propidium iodide and transfected with siCTL (Figure S4B), siM8-6a (Figure S4C) or siM8-7 (Figure S4D) for three days. A 7% decrease in the proportion of cells in G2/M phase was found in cells knocked-down with either siM8-6a or siM8-7 (Figure S4E). This confirms that sM8 KD-mediated p21 induction occurs mostly in quiescent cells and that this slight drop in G2/M cell proportion was most likely dependent on the full-length TRPM8 KD rather than on sM8 KD. A strong increase in the subG1 cell sub-population (Figure S4C and S4E) also confirmed a specific induction of apoptosis in sM8 KD cells. Altogether, our results demonstrate that sM8 KD triggers a concomitant induction of apoptosis and p21 expression, independently of cell cycle phase.

**Figure 4:**
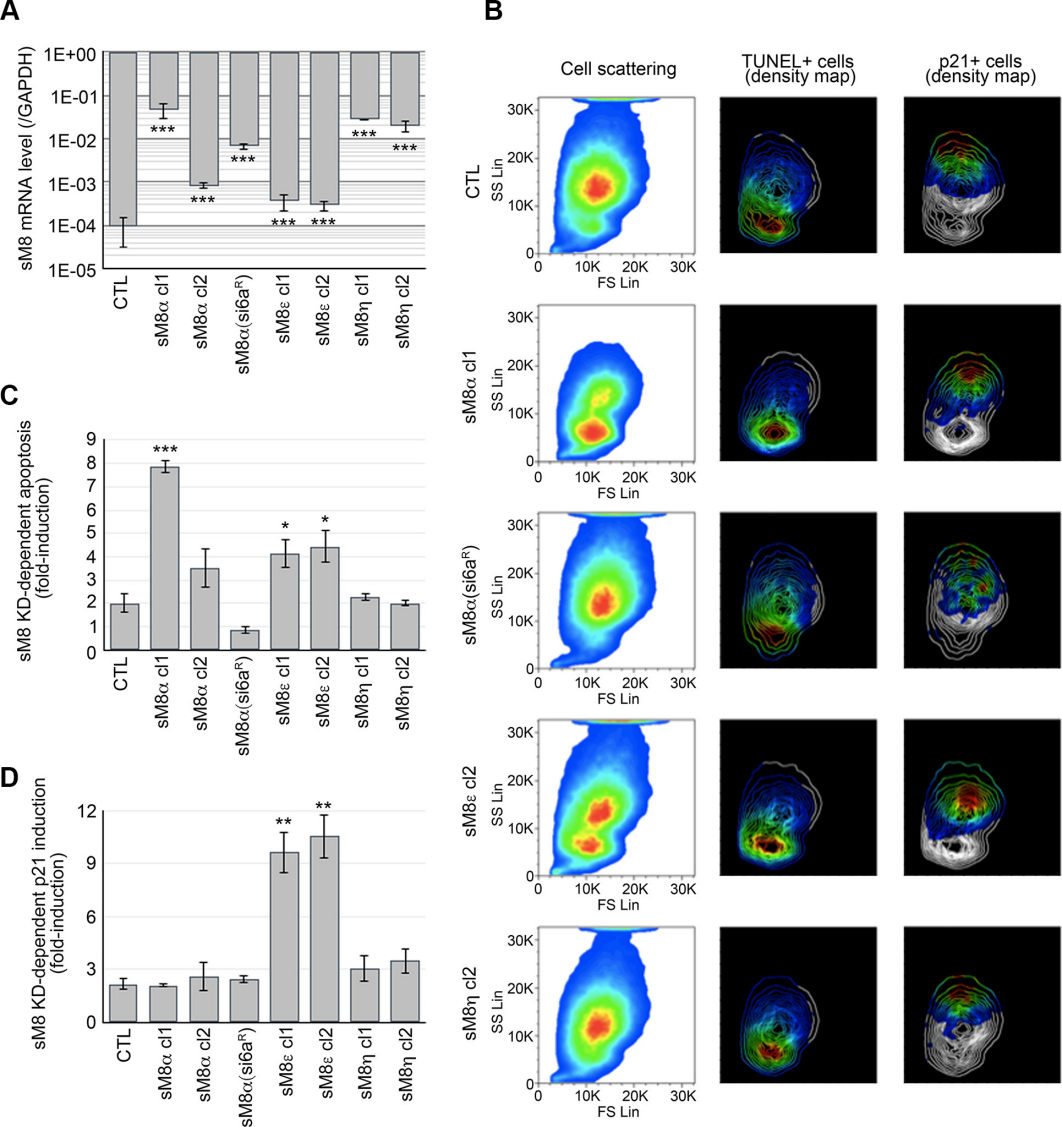
LNCaP C4-2b clones overexpressing a selective sM8 isoform become sensitized to either apoptosis or p21 induction following sM8 knockdown (**A**) LNCaP C4-2b clones stably overexpressing either sM8α or sM8ε or sM8η mRNA at different levels as reported by qPCR. 1 to 2 clones were analyzed per sM8 mRNA. CTL clones were transfected with empty pcDNA4 vector. sM8α(si6a^R^) mRNA has been mutated in order to resist to siM8(6a)-mediated silencing. (**B**) Overexpression of sM8 isoforms modifies the size and density of LNCaP cell population as represented in the pseudo-color cell scattering plots (left column). Cells were sorted out by flow cytometry and separated by size (Forward scattering light; FS Lin) and by granularity (Side scattering light: SS Lin). FACS analysis of apoptotic cells (TUNEL positive) and p21-immunolabeled cells is represented by the density pseudo-color plot in middle and right columns, respectively, while scattering of cells by size and granularity is represented by contour plot. Pseudo-color code represents expression levels from the lowest (blue) to highest (red). Each plot represents about 1 million cells. (**C**) Bar diagram plot shows the sM8 KD-mediated fold-induction of apoptotic cells calculated as the sM8 KD-induced apoptosis rate in sM8 overexpressing clones normalized by the apoptosis rate of wild type LNCaP C4-2. Control (CTL) was figured out as the average of apoptotic rates of two cell clones expressing the empty pcDNA4 vector. (**D**) Same as C but shows the fold-induction of p21 expressing cells. Experiments were performed three times independently. Values are expressed as Mean ± SD.

We have cloned five alternate sM8 mRNA and two splice variants, but their respective role in siM8-6a-mediated effect remained elusive since they were all knocked down simultaneously in our experiments. According to their mRNA and protein fingerprints in PCa, we developed C4-2b cell clones stably overexpressing sM8α, sM8ε or sM8η. A mutant sM8α clone, insensitive to siM8-6a mediated-KD, was also developed to control silencing specificity. As reported in Figure 4A, mRNA expression levels were measured by qPCR, as well as the efficiency of siM8-6a mediated KD (Figure S5A). In order to show their high diversity, the relative expression profiles of the three groups of TRPM8 isoforms in different prostate cancer cell lines are presented in the Figure S5B. The distribution of C4-2b cells overexpressing sM8s shows two distinct populations in flow cytometry in response to sM8 knockdown (Figures 4B and S5C): 1) apoptotic or 2) p21 positive cells. sM8 KD-induced apoptosis was potentiated in clones overexpressing sM8α in an mRNA concentration-dependent manner. Conversely, overexpression of the sM8α mRNA resistant to siM8-6a protected cells from sM8 KD-dependent apoptosis (Figure 4B and 4C). Surprisingly, sM8α overexpression did not sensitize sM8 KD-dependent p21 induction (Figure 4D). Overexpression of sM8ε sensitized C4-2b cells to apoptosis, though to a lesser extent than did sM8α, and drastically potentiated sM8 KD-dependent p21 expression. Finally, the non-coding sM8η mRNA antagonized neither sM8 KD-dependent apoptosis nor p21 induction in prostate cancer cells. In summary, knockdown of sM8α mRNA potently activates apoptosis while knockdown of sM8ε strongly induces p21. We thus wondered whether apoptosis and p21 induction mediated by sM8 knockdown implicated functional TRPM8 channels.

### Short TRPM8 (sM8) knockdown-mediated apoptosis is dependent on TRPM8 channel isoforms in LNCaP C4-2b

**Figure 5:**
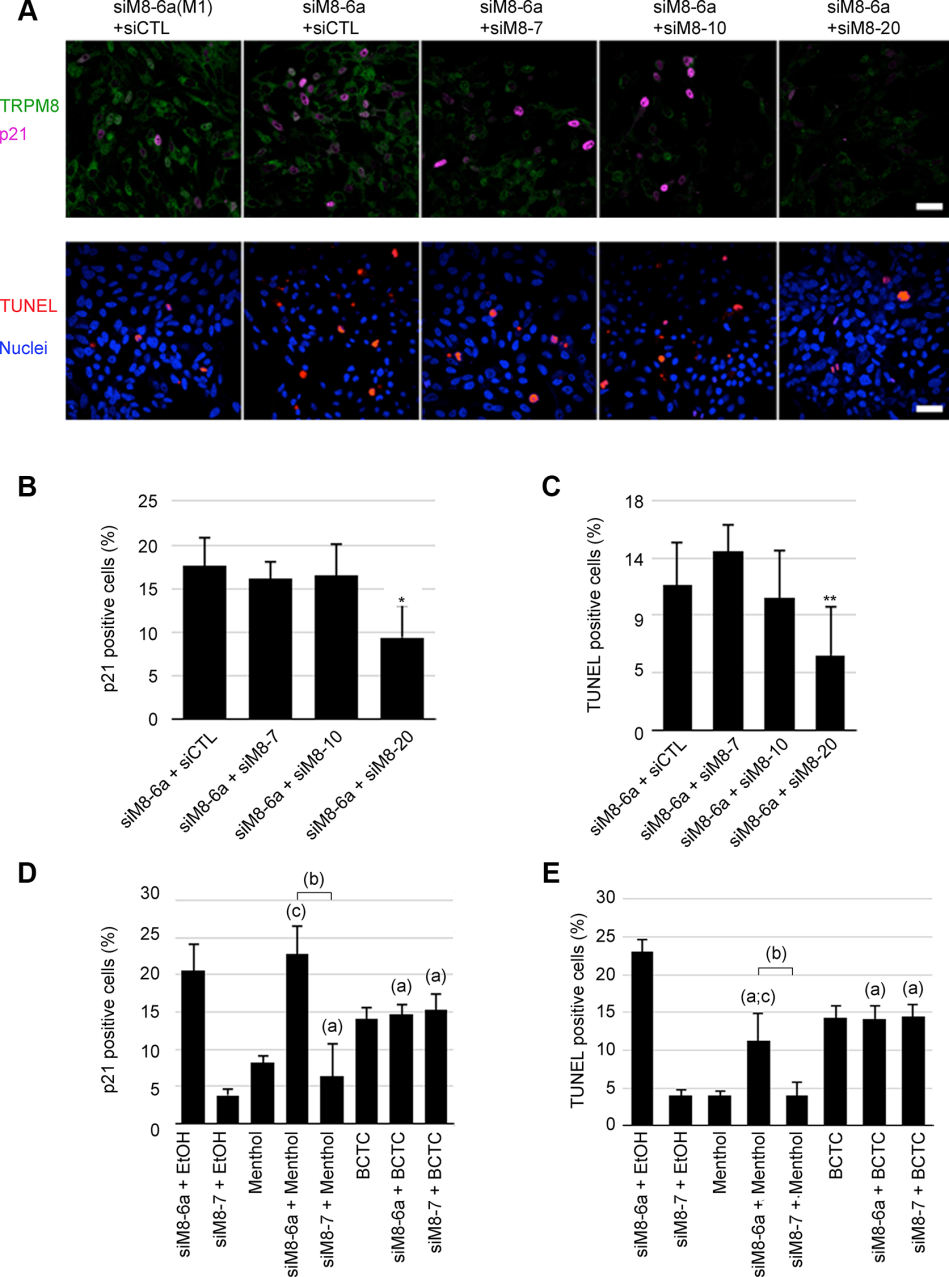
Apoptosis and p21 induction of sM8 knockdown C4-2b cells requires TRPM8 channel isoforms (4TM-TRPM8) (**A**) Immunocytofluorescence reports the concomitant expression of TRPM8 channels (green) and p21 (magenta), top row. The detection of apoptotic cells (bottom row) was achieved with TUNEL assay (red) in LNCaP C4-2 cultures after a 3-day transfection with siRNA, bottom row. Nuclei were counterstained with Dapi (blue). Scale bar: 10 μm. Cells subjected to a concomitant knockdown of the full-length TRPM8 channel (siM8-7 and siM8-10) and sM8 isoforms (siM8-6a), or a concomitant knockdown of the 4TM-TRPM8 channel (siM8-20) and sM8 isoforms were sorted for both p21 expression (**B**) and apoptosis by flow cytometry (**C**). Additions of TRPM8 agonist, menthol (500 μM, (**D**)), or a TRPM8 antagonist, BCTC (10 μM, (**E**)), in the medium have different effect on p21 induction and apoptosis of C4-2 cells transfected with siM8-6a. Experiments were performed three times independently. Values are expressed as Mean ± SD. (a): significantly different to sM8-6a+EtOH value, *p* < 0.05; (b): difference significant (*p* < 0.05) between sM8-6a and sM8-7 conditions with the same co-treatment; (c): difference significant (*p* < 0.05) with the treatment alone (menthol or BCTC).

We previously reported that sM8s are negative regulatory subunits of the full-length TRPM8 channel [14, 29]. Along this line, we postulated that sM8 could also modulate the activity of 4TM-TRPM8 which in turn could destabilize the ER-mitochondria calcium homeostasis - consequently leading to ER stress, oxidative stress and apoptosis. However, due to the localization of 4TM-TRPM8 in endomembranes, a direct demonstration of this sM8-mediated modulation of 4TM-TRPM8 activity was technically impossible in native condition of expression. We therefore carried out a concomitant transfection of siM8-6a (targeting sM8 and TRPM8) with either siM8-10 (targeting only TRPM8) or siM8-20 (targeting 4TM-TRPM8 and TRPM8) and quantified the size of cell populations engaged in apoptosis or expressing p21. As shown on Figure 5A, the transfection of LNCaP C4-2b cells with siM8-6a and either siM8-10 or siM8-20 drastically diminished the TRPM8 fluorescence level. However, only supplemental siM8-20 treatment (inducing an additional 4TM-TRPM8 KD) counteracted both sM8s KD-mediated p21 expression and apoptosis (Figure 5A–5C). We thus inferred that 4TM-TRPM8 isoforms were required for p21 and apoptosis induction in sM8 KD LNCaP C4-2b cells while full-length TRPM8 was not involved. To confirm the requirement of functional 4TM-TRPM8 channel in the sM8s KD-mediated induction of p21 and apoptosis, LNCaP C4-2b cells were treated with 25 nM of either siM8-6a or siM8-7 (control suppressing specifically TRPM8) and incubated for 3 days in presence of an agonist (500 μM menthol) or an inhibitor (10 μM BCTC) of TRPM8. We previously demonstrated that 4TM-TRPM8 is activated by menthol and inhibited by BCTC ([13]). Combined with sM8 KD, menthol did not potentiate p21 induction and, unexpectedly, diminished apoptosis (Figure 5D and 5E, respectively). Although, BCTC treatment increased the basal proportion of both p21 positive cells and apoptotic cells, it abolished sM8-6a effects (Figure 5D and 5E, respectively). Our results thus confirmed the requirement for functional 4TM-TRPM8 channels in the sM8 KD-mediated induction of p21 expression and apoptosis in LNCaP C4-2b cell line.

### sM8 knockdown also induces apoptosis and increases p21 in primary culture of human prostate cancer epithelial cells

Since next-generation sequencing has appeared, genomes and genetics aberrations of prostate cell lines have been extensively characterized, leading to questioning regarding their use as prostate cancer models. We therefore assessed the relevance of our findings in primary cultures of prostate cancer epithelial cells (PrPCa). Compared to prostate cancer cell lines (doubling time of LNCaP cell lines is about 32 hours), primary cultures proliferated slowly (doubling time was about 8 days) and consequently, in basal condition, 40% of cells expressed p21, while less than 2% were positive for Ki67. After two siRNA transfections within a 3-day interval, silencing efficiency reached only 50% - as compared to 90% in LNCaP cell lines – a caveat that would obviously attenuate siM8-6a-mediated effects. sM8 KD induced a significant increase in p21 and TUNEL positive cells, though, as expected, with less potency than in LNCaP C4-2b cell line (Figure S6A). Statistics revealed a 23%-increase in the p21 positive cells population (Figure S6B) which was further enhanced with menthol treatment (+39%) and reversed by BCTC treatment. Suppression or inhibition of 4TM-TRPM8 did not modify the proportion of proliferative cells (Figure S6C). Apoptotic cells were found to be 2.7-fold more abundant in the sM8 KD PrPCa cell population (Figure S6D). Menthol treatment of sM8 KD cells further increased the number of apoptotic cells by 30%. BCTC fully inhibited sM8 KD-mediated p21 induction and apoptosis (Figure S6B and S6D). Altogether, these results support the idea that sM8 KD promotes p21 expression and apoptosis in PrPCa as it does in LNCaP C4-2b cell line.

### sM8 mRNAs/isoforms differentially regulate the expression of TRPM8 and 4TM-TRPM8 mRNA and alters Ca^2+^ homeostasis

**Figure 6:**
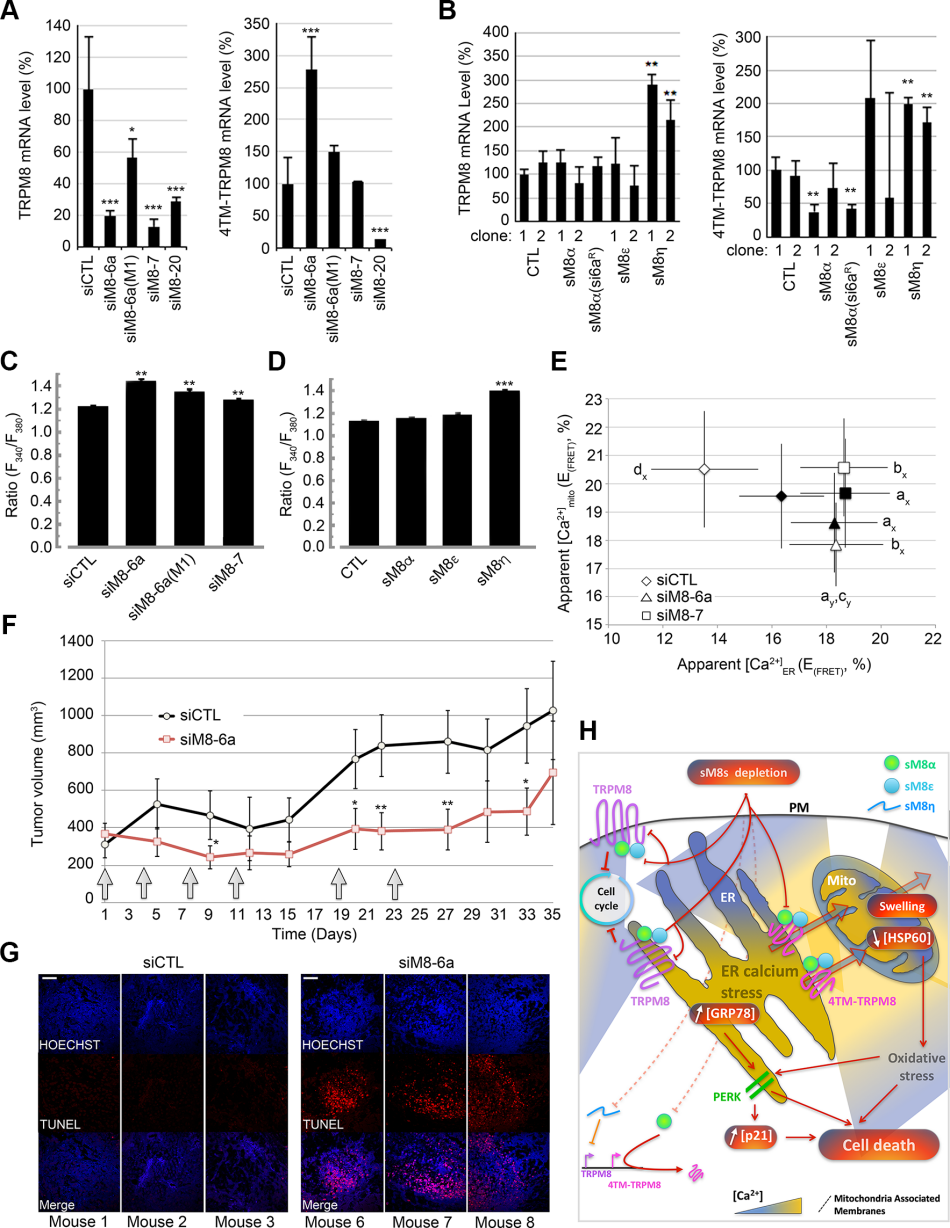
sM8 isoforms participate to the regulation of the level of expression of TRPM8 proteins and to Ca2+ concentration in both ER and mitochondria **(A)** Bar diagram plots show expression level of TRPM8 mRNA (left panel) and of 4TM-TRPM8 (right panels) as measured by qPCR. Knockdown of sM8 isoforms with siM8-6a (left panel, calculations described in Methods section) induces expression of 4TM-TRPM8 (right panel), although neither mutant siM8-6a(M1) nor specific KD of TRPM8 channel, siM8-7, modify 4TM-TRPM8 expression. (**B**) mRNA levels of TRPM8 (left panel) and 4TM-TRPM8 (right panel), measured by qPCR, in C4-2b clones overexpressing specific sM8 isoforms are presented in bar diagram plots. (**C**) Apparent [Ca^2+^]_cyto_ was determined as the ratio of fura2-AM fluorescence normalized by its value at the origin of experiment. Cells were previously transfected with 50 nM of siRNAs targeting luciferase (si Luc, *n* = 387) or TRPM8 (siM8-6a, *n* = 348; siM8-6a(M1), *n* = 184 and siM8-7, *n* = 345) for 3 days. Experiments were carried out in the absence of extracellular calcium, (**D**) As C, in either control LNCaP C4-2b clones (CTL), *n* = 344; or in LNCaP C4-2b clones overexpressing sM8 α, *n* = 307; sM8 ε, *n* = 302; sM8 η, *n* = 280. (**E**) Graph plot shows the FRET efficiency, E_(FRET)_, of ER (ERCam) and mitochondria (MitoCam) targeted Cameleon biosensor in CTL LNCaP C4-2b cells (black markers) or in sM8α -overexpressing LNCaP C4-2b clone 1 (open markers). Cells were previously transfected with CTL siRNA (lozenge, 40 cells), siM8-6a (triangle, 40 cells) or siM8-7 (square, 40 cells). The two latter conditions report the effect of 1) the concomitant suppression of full-length TRPM8 and sM8s, 2) the suppression of full-length TRPM8 alone. Experiments were performed independently three times. Values are expressed as Mean ± SD. Statistical significance was assessed with One-way ANOVA and accepted for *p* < 0.05; a: different from siCTL in *wt* C4-2b cells, b: different from siCTL in sM8α-overexpressing C4-2b cells, c: difference between siM8-6a and siM8-7 and d: difference between *wt* and sM8α-overexpressing C4-2b. (**F**) Tumor growth of sM8 α-overexpressing LNCaP C4-2b cells grafted, subcutaneously, in mice. After tumor implantation was confirmed, six injections (gray arrows) of siRNA (CTL or siM8-6a) were achieved in 2 series of 5 mice. Tumor volume was then calculated regularly over the 35 days of the experience. Graph plot shows the tumor volume (Mean ± SD). After 35 days, mice were sacrificed and tumors were collected for TUNEL assay. (**G**) Shows the detection of apoptotic cells (labeled with TUNEL-TMR kit) in the tumors of 3 mice injected with siCTL or 3 mice injected with siM8-6a. All cell nuclei within the tumors were stained with Hoechst. Scale bar: 50 μm. (**H**) Schematic representation of the role of TRPM8 isoforms in the sM8 KD-mediated molecular cascade inducing apoptosis of prostate cancer cells. The concomitant knockdown of TRPM8 and sM8s induces perturbations in the ER-mitochondria Ca^2+^ homeostasis, down regulation of TRPM8 transcription and an increase in 4TM-TRPM8 transcription (bottom left corner). This, in turn, induces ER stress (increased GRP78 expression; PERK pathway activation), and cell cycle arrest, mitochondria oxidative stress (reported by the decrease of HSP60 expression). These mechanisms likely lead to mitochondrial permeability transition (swelling) accompanied by a massive leak of Ca^2+^ from mitochondria to cytosol. Altogether these mechanisms participate to the induction of apoptosis. Mito: mitochondria, PM: Plasma Membrane, ER: Endoplasmic Reticulum.

Finally, we aimed at understanding whether knockdown of sM8 isoforms could interfere with Ca^2+^ homeostasis and TRPM8 channel expression. Negative feedback loops exerted by small RNA/protein on their mother-gene transcriptional activity have been reported for different genes (for review, see [30]). By performing qPCR with pairs of primers matching either the full-length TRPM8 sequence (exons 7 to 8) or all TRPM8 channel-forming TRPM8 isoforms (exon 19 to 21), we measured and compared the expression of full-length TRPM8 vs. 4TM-TRPM8 isoform while suppressing sM8s in LNCaP 4-2b cells. As expected, expression levels of mRNA coding 4TM-TRPM8 isoform decreased within cells transfected with siM8-20 (Figure 6A, right panel) and was kept unchanged after transfection with siM8-7. Unexpectedly, a strong increase in 4TM-TRPM8 mRNAs (2.5 fold) was detected in sM8s KD cells (siM8-6a), while a smaller effect was observed in cells transfected with the mutant siM8-6a(M1). Since sM8-6a has no complementation with the 4TM-TRPM8 sequence, this induction of 4TM-TRPM8 suggests a feedback regulatory loop caused by sM8 expression. To confirm this, we measured TRPM8 and 4TM-TRPM8-coding mRNAs expression in C4-2b clones overexpressing sM8s. As reported in the Figure 6B, both full-length TRPM8 and 4TM-TRPM8 expression increased in cells overexpressing the non-coding sM8η mRNA. However, a decrease in 4TM-TRPM8 expression was found in cells strongly overexpressing sM8α (right panel). This suggests that 4TM-TRPM8 induction in sM8 KD cells could be related to the specific silencing of sM8α mRNA. Since we previously reported that the full-length TRPM8 channel and its channel isoforms were functional in ER membranes [9], we hypothesized that modifications in their expression or activity after sM8 KD could modify Ca^2+^ homeostasis. We thus quantified steady-state Ca^2+^ concentration ([Ca^2+^]_cyto_) in cytosol, ER and mitochondria of LNCaP C4-2b cells transfected with siRNAs targeting TRPM8 and in LNCaP C4-2b clones overexpressing various sM8 isoforms. A 2-day suppression of sM8s and full length TRPM8 (siM8-6a) induced a concomitant increase in [Ca^2+^]_cyt_ (Figure 6C) and [Ca^2+^]_ER_ (Figures 6E and S7A), while [Ca^2+^]_mito_ decreased (Figures 6E and S7C).

In mitochondria, apoptosis can be divided in 3 phases: 1) Ca^2+^ overload, inducing 2) cyclophilin D-mediated mitochondrial permeability transition– consequently triggering the loss of mitochondrial membrane potential and matrix Ca^2+^ leak [31] and 3) mitochondria outer membrane permeabilization leading to the release of pro apoptotic proteins. To confirm that sM8s KD-mediated decrease in [Ca^2+^]_mito_ was a marker of the intermediate step leading to death of mitochondria, we performed transmission electron microscopy. Images revealed swollen mitochondria, cristae disorganization and mitophagy, specifically in siM8-6a-treated cells (Figure S7E). This suggests that the total Ca^2+^ buffering compartment in mitochondria could be decreased in sM8-KD cells and would thus explain why sM8 KD cells showed a reduced Ca^2+^ content. Conversely to the effect of sM8 KD, the overexpression of sM8α in LNCaP C4-2b cells decreased ER Ca^2+^ content (Figure 6E and S7B) without altering [Ca^2+^]_cyto_ (Figure 6C) and [Ca^2+^]_mito_ (Figure 6E). By contrast, the overexpression of the non-coding sM8η mRNA induced a significant increase in [Ca^2+^]_cyto_ (Figure 6D). The overexpression of either sM8ε or sM8η did not shift [Ca^2+^]_ER_ (Figure S7B). These results thus suggest that the modification in ER calcium content is correlated to sM8α expression level. However, one can note that the down regulation of full length TRPM8 channel also increased both [Ca^2+^]_cyt_ and [Ca^2+^]_ER_ (Figures 6E and S7D) without modifying [Ca^2+^]_mito_ (Figure 6E). We conclude that sM8 KD triggered indirect modifications in the ER-mitochondria Ca^2+^ homeostasis that could participate to prostate cancer cell death.

Since both sM8 KD-mediated cell death, *in vitro*, and sM8 KD-induced modifications in Ca^2+^ homeostasis are potentiated in cells overexpressing the sM8α isoform, we next checked whether it also increased tumors sensibility. Nude mice were subcutaneously grafted with sM8α overexpressing C4-2b cells. After tumor implantation, mice were subjected to six injections, of either siCTL or siM8-6a, over a period of 23 days. Results presented in the Figure 6F demonstrated an obvious decrease of tumor growth that is mainly carried out by a massive apoptosis occurring inside the tumors as revealed by TUNEL and Hoechst staining (Figure 6G).

## DISCUSSION

In 2011, we characterized a new short TRPM8 mRNA (sM8α) and its splice variant (sM8β) and we demonstrated that both encoded two cytosolic sM8 proteins: sM8-6 and sM8-18 which behave as negative regulatory subunits of the full-length TRPM8 channel in plasmalemma [14, 29]. In the present study, we have isolated 3 additional alternate mRNAs and 2 splice variants. We show here that 5 mRNAs encode 4 proteins while 2 other mRNAs are likely non-coding. Though long non-coding mRNAs were originally thought to be products of either mRNA degradation or defective transcription, recent evidences have revealed that they might regulate the expression of their mother-gene (for review, see [30]). Along this line, we demonstrate firstly that the expression level of the non-coding sM8η mRNA is positively correlated to the expression of full-length TRPM8 mRNA. Secondly, the expression levels of sM8α mRNA and 4TM-TRPM8-coding mRNA are inversely correlated, while sM8ε mRNA do not regulate at all the expression of full-length TRPM8 or 4TM-TRPM8 channels. Altogether, these results suggest specific back-loop regulations of TRPM8 and 4TM-TRPM8 transcriptions by sM8.

Our results indicate an essential role of TRPM8 channels in sM8 KD biological effects. However, full-length TRPM8 and 4TM-TRPM8 channels might participate to different outcomes. Indeed, as reported previously by Valero et al. [17], we here confirm that suppression of full-length TRPM8 correlates with a slight decrease in the cell-cycling population that could explain the decrease in cell growth we observed. Conversely to full-length TRPM8 silencing, 4TM-TRPM8 knockdown suppressed sM8 KD-mediated p21 induction and apoptosis. Altogether, our results emphasize specific roles of full length TRPM8 (cell cycle), 4TM-TRPM8 (cell cycle arrest via p21 and apoptosis), sM8α (apoptosis) and sM8ε (p21 induction and apoptosis). Although the understanding of the underlying molecular mechanisms remains incomplete and should be addressed in further studies, our preliminary data indicate that Ca^2+^ homeostasis between ER and mitochondria is involved.

In the present study, single cell analysis has revealed that the concomitant knockdown of full-length TRPM8 and sM8 induced 1) an increase in [Ca^2+^]_ER_ paralleled by 2) a decrease in [Ca^2+^]_mito_. Our data revealed that the former could be reproduced by the specific suppression of full-length TRPM8 alone. However, the overexpression of sM8α isoform in C4-2b cells decreased their ER Ca^2+^ content. These results altogether suggest that both full-length TRPM8 and sM8α are key regulators of [Ca^2+^]_ER_. Increased [Ca^2+^]_ER_ could participate to apoptosis but may not be sufficient by itself. Indeed, others and we have previously demonstrated that cell death is a finely tuned mechanism linked to ER Ca^2+^ stores (for review see [2, 32–34]). In prostate cancer cells overexpressing the anti-apoptotic Bcl-2 protein, we reported that ER Ca^2+^ content was lowered [35]. Reciprocally, an increase in [Ca^2+^]_ER_ could sensitize cells to apoptosis (for review see [36]) by exposing mitochondria to overloading Ca^2+^ amounts promoting the opening of the permeability transition pore (PTP). However, we have found here that sM8s KD but not TRPM8 KD triggers a decrease in [Ca^2+^]_mito_ and induces apoptosis. This suggests that although an increase in [Ca^2+^]_ER_ could participate to sM8 KD-mediated apoptosis, it is not sufficient by itself.

We previously demonstrated that the preferential localization of 4TM-TRPM8 isoform in keratinocytes ER membranes was a key determinant of the regulation of Ca^2+^ ER/Mito homeostasis by cold or menthol [13, 37]. On the other hand, we have reported that sM8α-encoded sM8-18 protein is a negative regulatory element of the full-length TRPM8 channel [14, 29]. It could have thus been expected that sM8 KD should increase [Ca^2+^]_mito_ and decrease [Ca^2+^]_ER_ through both the induction of 4TM-TRPM8 expression and the release of 4TM-TRPM8 inhibition by sM8s. However, since sM8 KD is concomitant to apoptosis, it is not trivial to measure sM8 KD-dependent changes in [Ca^2+^], independently of apoptosis-associated changes in [Ca^2+^]. Indeed, a decrease in [Ca^2+^]_mito_ can result from a massive decrease in Ca^2+^ tunneling from ER to mitochondria, but can also be the consequence of the loss of mitochondrial potential and PTP opening during apoptosis (for review see [38, 39]). Along this line, we noticed that: firstly, the concomitant increase in both [Ca^2+^]_cyto_ and [Ca^2+^]_ER_ should favor calcium reuptake by living mitochondria and thus lead to an increase in [Ca^2+^]_mito_ (for review see [40]). Secondly, siM8-6a transfected cells showed a significant number of swollen mitochondria likely reflecting PTP opening.

Calcium is an essential component of the regulation of mitochondrial bioenergetics and oxidative stress (for review, see [36, 38]). The chaperone HSP60 is an essential protein involved in the protection of mitochondria against oxidative stress and its mutation causes diseases such as the hereditary spastic paraplegia [41]. HSP60 action includes correct folding of mitochondrial proteins like the manganese superoxide dismutase [42], which is the major superoxide anion-detoxifying enzyme in the mitochondrial matrix. Superoxide anion is a byproduct of the respiratory chain complexes I and III activities [34] which can be either transformed in H_2_O_2_ or in peroxinitrites, two highly reactive oxygen species, that in turn increase lipid peroxidation and protein S-nitrosilation [38, 43, 44]. HSP60 downregulation was found to sensitize cells to oxidative stress and thus to potentiate cell death in several cell types [42, 45, 46]. Since our results show a strong decrease in HSP60 expression level, it is therefore likely that sM8 KD-mediated cell death involves oxidative stress and protein unfolding in mitochondria. Along this line, we previously demonstrated that an increased activity of 4TM-TRPM8 isoforms was correlated to an increased concentration of both ATP and superoxide in keratinocytes mitochondria [13]. Besides, ER possesses a unique capacity to sense and respond to various stress stimuli such as the accumulation of excessive or misfolded protein (UPR pathway), deprivation of glucose, calcium fluctuations, oxidative stress. Three ER stress sensors are described: IRE1α (inositol-requiring protein-1α), PERK (protein kinase RNA (PKR)-like ER kinase), and ATF6 (activating transcription factor 6). They are involved in several steps of this survival process (*i.e* increasing production of additional chaperone proteins (i.e. GRP78) to accelerate protein folding and detoxification, reduce protein translation, cell cycle…) but if the stress burden becomes too important, cell death is unavoidable (for review, read [18, 19]). Among these three pathways, PERK has been characterized as the oxidative stress sensor in ER and is required at the ER-mitochondrial contact sites to convey apoptosis after ROS-based ER stress [47]. Strikingly, in our study, we have demonstrated that PERK was required for sM8 KD-mediated apoptosis of prostate cancer cells. As depicted in the Figure 6H, we thus propose a complex regulation process induced by sM8 KD which involves perturbation of the ER-mitochondrial Ca^2+^ homeostasis, oxidative stress characterized by the down regulation of HSP60, and activation of PERK pathway in ER.

Finally, our *in vivo* experiments confirm that targeting sM8 isoforms could be an efficient anti-cancer strategy in sM8-expressing tumors. However, we concluded that the higher the sM8/4TM-TRPM8 ratio was, the more efficient the treatment would be. Besides, the discrepancy in the treatment efficiency between CTL C4-2b and sM8α-overexpressing C4-2b grafts could be related to the proportion of cells expressing sM8α/ε above a responsive threshold. Indeed, the range of CTL C4-2 cells ongoing sM8 KD-mediated apoptosis was comprised between 5 to 20%, which could reflect the proportion of cells expressing sM8α/ε mRNAs. In comparison, all cells from sM8α-overexpressing tumors expressed sM8α mRNA, which led to 50–60% of cells dying after sM8 KD.

In conclusion, we believe that this sM8-based strategy should be incorporated to a multiplex approach targeting different cellular and molecular pathways in order to restrict escape pathways and thus cancer relapse. *In vivo* gene silencing has emerged in the past decade as a potential strategy to trigger cell death inside tumors. However, improvement of siRNA vectorization must still be achieved to improve their delivery into the tumor.

## MATERIALS AND METHODS

### Cell lines culture

The LNCaP and PC-3 cell lines were grown in complete RPMI medium 1640 (Gibco).

### Primary culture of human prostate cancer epithelial cells (PrPCa)

Primary cultures of human prostate epithelial cells were prepared as described in Supplementary Information.

All experiments on human tissues were performed according to the “CP 01/33” regulations issued by the “Comité Consultatif de Protection des Personnes dans la Recherche Biomedicale de Lille” (CCPPRB).

### Transfection

Cells were transfected with HiPerfect (Qiagen) once and experiments were performed 3 days after transfection. PrPCa were transfected twice with a 3-day recovery period between transfections and experiments were performed 3 days after the second transfection. siRNAs are listed in Table S1.

### Group suppression of TRPM8 isoforms

We postulated that group suppression of TRPM8 isoforms could result in different cellular and molecular phenotypes. We also assumed that a subtractive comparison should reveal information about the biological function of a specific group of TRPM8 isoforms. For instance, while siM8-6a silenced both sM8 and TRPM8, siM8-7 silenced only TRPM8. We thus expected to specifically correlate the biological variations between both siRNAs to sM8 knockdown (KD). To address this strategy, we developed a series of siRNAs targeting different TRPM8 exons spanning over the TRPM8 gDNA (sequences are presented in Table S1). siRNAs were labeled in reference to the TRPM8 exons they were targeting (i.e siM8- exon number). Efficiency of siRNA on TRPM8 silencing was measured in TRPM8-35 inducible HEK cells transfected with 50 nM of siRNA for 48 h (Figure S1A). qPCR (Figure S1A) and western-blot (Figure S1B and S1C) experiments validated two siRNAs targeting TRPM8 (siM8-7 and siM8-10), three siRNA targeting the sM8s in addition to TRPM8 (siM8-4b, siM8-6a and siM8-6a.2) and one siRNAs targeting TRPM8-35 in addition to TRPM8 (siM8-20) (Figure 1A).

### Subcutaneous xenograft

Six-weeks old male swiss nude mice (Charles River Laboratories, France) were injected with LNCaP C4-2b cells. Six million cells were injected into one flank of each mouse. Cells were prepared in a mixture composed of 50% PBS and 50% BD-Matrigel^®^ (BD Bioscience, France). Tumors were measured twice a week using a caliper and animals were sacrificed 12 weeks after injection unless the mice had to be sacrificed earlier if the total tumor size reached 10% of the animal weight. 7.5 μg of siRNA were injected i.p. with a minimum of 3-day interval as presented in the Figure 6F.

Tumor volume was assumed to be a spheroid and calculated using the formula: Vi = A^2^ *C*π/6; where Vi: volume inside the spheroid, A: equatorial diameter and C polar diameter.

On the day of sacrifice, tumors were weighed and divided for further immuno-histochemical, western blot and RT-PCR experiments. At least 10 animals per condition were used. *In vivo* experiments were conducted on mice according to the agreement provided by the local ethical comity (protocol CEEA 202012).

### 5′-RACE PCR

5′ alternate extremities of short TRPM8 isoforms (sM8) were cloned with SMART RACE-PCR, following the manufacturer procedures (Clonetech).

### Cloning of sM8 mRNA

Specific primers, based on RACE sequences, were designed and used to amplify cDNA with Taq Gold polymerase from LNCaP mRNA, prepared as described above. Amplicons were ligated in pGemTeasy vector (Promega). After PCR screening, clones were subjected to sequencing. Both TRPM8 splice variants were then inserted in pcDNA4.TO.A (Invitrogen).

### Real-time PCR

Real-time quantitative PCR was performed on a Cfx C1000 system (Biorad) with SsoFast^™^ EvaGreen^®^ Supermix (Biorad).

### Immunoblotting

An SDS-page was performed using 25 μg of total protein loaded into a 10% polyacrylamide gel. After electrophoresis, proteins were transferred to a nitrocellulose membrane using a semi-dry electroblotter (Bio-Rad). The membrane was processed for chemiluminescence detection using Luminata Forte Western HRP Substrate (Millipore) according to the manufacturer's instructions. The primary antibodies were: rabbit anti-TRPM8 (Ab109308, Abcam, detecting the pore region of TRPM8), rabbit anti-HA tag (Sc-805, Santa Cruz) and goat anti-GA3PDH (Sc-20357, Santa Cruz).

### Immunocyto Fluorescence

The primary antibodies used were: rabbit anti-TRPM8 (Ab109308, Abcam), mouse anti-p21 (Clone SX118, Dako), rabbit anti- cytokeratin 14 (PRB-155P-100, Covance), rabbit anti- cytokeratin 5 (PRB-160P-100, Covance), mouse anti-cytokeratin 18 (Sc-51582, Santa Cruz) and mouse anti-Vimentin (Clone V9, Dako).

### Apoptosis assay

Briefly, according to the manufacturer's protocols (TNR Red Roche), formalin-fixed and paraffin-embedded tissues tumor slices (6 μm) are incubated with PBS-G at 37°C in a humidified dark chamber. After TUNEL staining reaction (30 min, 37°C) in humidified dark chamber, slices are washed twice with PBS-G. Finally slices are stained with Hoechst/PBS (1/5000). Staining is visualized by LSM 700 confocal imaging system.

### Flow cytometry

Flow cytometry was performed with a CyAn^™^ ADP Analyser.

For analysis of immunolabeled cell population, mouse anti-p21 (Clone SX118, Dako) primary antibody was diluted in 100 μl PBS-BT at 1/200 and incubated with cells at ambient temperature for 1 h. After a first quick wash in PBS, a second wash was done at RT for 30 min. Cells were incubated with rabbit anti-Ki67 antibody coupled to FITC (Ab27619, Abcam) and/or secondary antibodies specific to the host animal of the primary antibodies, antirabbit IgG coupled to Dye Light-488 (Jackson ImmunoResearch; dilution 1/2000) or anti-mouse IgG coupled to Alexa Fluor 647 (Jackson ImmunoResearch; dilution 1/4000) at RT for 30 min. After two PBS washouts for a total incubation time of 30 min at ambient temperature, cells were suspended in 500 μl of PBS and then analyzed.

For TUNEL (terminal deoxynucleotide transferasemediated dUTP—biotin nickend labelling) experiments, cells were pelleted and then suspended in 100 μl of labeling solution (TUNEL-TMR red, Roche) at room temperature for 30 min. Cells were washed once in PBS at ambient temperature for 10 min.

For cell cycle analysis, cells were suspended in 250 μl PBS/4% BSA/0, 1% Triton ×100 with Ribonuclease A (200 μg/ml) and incubated at room temperature for 15 min. After addition of 250 μl of PBS containing 30 μg/ml of propidium iodide, cells were further incubated at room temperature for 30 min to 1 hour. Data were analyzed with Flow Jo software (version 8.7).

### Viability assay

CellTiter 96^®^ AQ_ueous_ Non-Radioactive Cell Proliferation Assay (Promega) was used to determine the number of viable cells each day.

### Wide-field Ca^2+^ imaging

Calcium imaging experiments have been performed as described previously [10].

### Morphology analysis by transmission electron microscopy

For morphology analysis, cells were fixed in 2.5% glutaraldehyde dissolved in 0.1 M cacodylate buffer and were post-fixed in 1% osmium tetroxide in the same buffer. After acetonitril dehydration, the pellets were embedded in Epon. Serial thin sections (90 nm) were cut using a Leica UC7 ultramicrotome and collected on 150 mesh hexagonal barred copper grids. After staining with 2% uranyl acetate prepared in 50% ethanol and incubation with a lead citrate solution (Reynolds), sections were observed on a Hitachi H-600 transmission electron microscope at 75 kV.

### Time domain-fluorescence lifetime imaging microscopy (TM-FLIM)

For live-cell imaging, cells were placed on 35 mm glass bottom dishes (MatTek Corporation, USA), filled with L-15 medium without phenol red (Life technologies), and incubated at 37°C in a thermostatic chamber (Life Imaging Services, Switzerland). D1ER [48] and 4mtD3cpv [49] Cameleon biosensors were transfected in LNCaP C4-2b cells and used for determining calcium free concentration in either ER or mitochondria. FLIM was performed with a Leica TCS SP5 X confocal head (Leica Microsystems, Germany) with a pulsed diode laser, PDL 800-B (Pico Quant GMBH, Germany), delivered 40 MHz repetitive rate pulses at 405nm. Photons were detected by a TCSPC detector (HydraHarp 400; Pico Quant GMBH, Germany). Arrival time of single photons was measured with Sym PhoTime software (Pico Quant GMBH, Germany) while image were taken with LAS AF software (Leica Microsystems, Germany). Fluorescence lifetime of the donor of fluorescence was determined by the Phasor plot method using a homemade software: MAPI [50].

Since fluorescence lifetime is independent of the concentration of the fluorescence emitter, the FRET-FLIM measurement of a biosensor limits artifacts due to the variation of concentration in a single cell or between different cells. FRET efficiency value, namely E_(FRET)_, was Figured out from eq(1)

Eq(1) E_(FRET)_= 1−(τ_DA_/τ_D_) and directly correlates to the proportion of Ca^2+^-bound Cameleon, then it consequently correlates to steady-state [Ca^2+^].

### Data analysis

Each experiment was repeated at least three times and the results were expressed as mean ± S.E.M. The data were analyzed and graphs plotted using Origin 5.0 software (Microcal, Northampton, MA). InStat3 (GraphPad Software Inc, San Diego, USA) was used for statistical analysis and mean values were compared using either unpaired *t* test with Welch's corrected test (2 groups) or One-way ANOVA with Dunnett multiple comparison post-test (≥ 3 groups). Statistical significance was denoted by (*) for *p* < 0.05, (**) for *p* < 0.01 or (***) for *p* < 0.001. For ethical reasons, the minimum group size of animal experiments was limited to 9. Thus, statistical significance was calculated by a two-tail *t*-test with a size effect of 1.5 (low sensitivity), a power of 0.8 (high specificity), and α < 0.05 (σ of all groups was assumed to be 1 for experimental design).

Full descriptions of procedures are presented in Supplementary Information.

## SUPPLEMENTARY MATERIALS TABLES AND FIGURES


