# Supplementary material for: Targeting of short TRPM8 isoforms induces 4TM-TRPM8-dependent apoptosis in prostate cancer cells
Source: Oncotarget. 2016 Apr 9;7(20):29063–80. doi: 10.18632/oncotarget.8666 (PMC5045378; doi:10.18632/oncotarget.8666)
Supplement: Supplementary file 1 [file oncotarget-07-29063-s001.pdf]

Targeting of short TRPM8 isoforms induces 4TM-TRPM8-dependent apoptosis in prostate cancer cells

Supplementary Materials

MATERIALS AND METHODS

Cell lines culture

The LNCaP, DU145 and PC-3 cell lines were purchased from the American Type Culture Collection (ATCC). LNCaP C4-2b cell line was a generous gift from Dr. F. Cabon, Paris. Cells were amplified in RPMI medium 1640 (Gibco®) supplemented with 10% fetal calf serum (FCS) and kanamycin (100 µg/ml). For experiment, cells were cultured in RPMI medium 1640 (Gibco®) supplemented with 2% FCS, 1 mM Sodium pyruvate, 1.5 mM $CaCl_2$, and kanamycin (100 µg/ml).

Primary culture of human prostate cancer epithelial cells (PrPCa)

Human prostate tissue specimens were obtained from resection surgeries performed on clinical indications in the Urology Department at l'Hôpital St. Philibert (Lille, France). All specimens came from patients who had not received anti-androgen therapy. In addition, all specimens were diagnosed by an anatomopathological examination. After patient surgery, the connective tissues were eliminated and the epithelial nodules were cut into small fragments of about 1–2mm^3 in a 100 mm diameter dish filled with 3ml of RPMI 1640 medium. Fragments of tissue were washed in clean RPMI medium prior to their transfer in culture dishes. About 7 explants were put in BD *Primaria*™ 100 mm Cell Culture Dishes with 1 to 1.5 ml of basal PrPCa medium for 2-3 days – to let the explants adhere on the dish surface. Thereafter, extra medium was added, and renewed twice a week for 4 to 5 weeks or until cells outspreading from explants reached confluence. Before carrying out experiments, cells were detached with a 30 min incubation in 9 ml of a PBS solution containing 0.5 mM EDTA. One ml of trypsin was then added for a further 5 min-incubation. Cells were collected and centrifuged prior to their resuspension in basal PrPCa medium. After a 1-day recovery period, cells were transfected and induced in the induction PrPCa medium. Basal PrPCa medium was: Keratinocyte-SFM medium (Gibco®) supplemented with EGF, BPE and kanamycin (100 µg/ml). Induction PrPCa medium was: Keratinocyte-SFM medium (Gibco®) supplemented with EGF, BPE, kanamycin (100 µg/ml), 2% FCS and 1.7 mM $CaCl_2$.

All experiments on human tissues were performed according to the "CP 01/33" regulations issued by the "Comité Consultatif de Protection des Personnes dans la Recherche Biomedicale de Lille" (CCPPRB).

Transfection

Cells were transfected with plasmids using nucleofector technology (Lonza). Briefly, 1 million cells were transfected with 2 µg of total vectors and then seeded on precoated dishes with polylysine. siRNA (25 nM) transfection was performed with HiPerfect (Qiagen) as described by the manufacturer. Cell lines were transfected once and experiments were performed 3 days after transfection. PrPCa were transfected twice with 3 days recovery between transfections and experiments were performed 3 days after the second transfection. siRNAs are listed in Table S1.

5′-RACE PCR

5′ alternate extremities of short TRPM8 isoforms (sM8) were cloned with SMART RACE-PCR, following the manufacturer procedures (Clonetech). Briefly, total mRNA of LNCaP cells were treated with DNAse 1 for 15 minutes prior to phenol/chloroform purification. After ethanol precipitation of mRNA, first strand cDNA were synthesized with Prime script Reverse Transcriptase (Takara) from 2 µg of mRNA using SMART oligonucleotides. To clone sM8 mRNA, 5′-RACE-PCR was performed with phusion polymerase (Finzymes) for 35 cycles with TRPM8 specific backward oligonucleotide: CACAATATTCTCCTCTGAACTCCT. After cleaning up PCR mix on columns (Nucleospin extract II, Macherey Nagel), nested-PCR was performed on 1 µl of RACE purified DNAs with phusion polymerase. Amplicons were visualized on agarose gel with syber green on a Dark reader, blue light table (Ozyme). After excision of bands, DNA was recovered with Nucleospin extract II (Macherey Nagel). Terminal adenylation of DNAs was then performed with Taq gold polymerase prior to cloning PCR products in pGem-T easy vector (Promega). Colonies were controlled by EcoRI digestion and positive clones were submitted to sequencing.

Cloning of sM8 mRNA

Specific primers, based on RACE sequences, were designed and used to amplify cDNA with Taq Gold polymerase from LNCaP mRNA, prepared as described above. Amplicons were ligated in pGemTeasy vector (Promega). After PCR screening, clones were subjected to sequencing. Both TRPM8 splice variants were then inserted in pcDNA4.TO.A (Invitrogen).

HA-tagged fusion proteins

The forward PCR oligonucleotide includes the Not I restriction site and conserves the endogenous Kozak sequence. The backward PCR oligonucleotide incorporates a substitution of the STOP codon prior to the HA sequence followed with a Xho I restriction site. After PCR amplification, PCR products were digested overnight at 37°C, and finally subjected to agarose gel purification and DNA recovery (Wizard®SV gel, Promega). pcDNA4.TO.A vector has been opened with both Not I/Xho I before dephosphorylation with Antarctic Phosphatase (New England Biolabs). Restricted PCR products and dephoshorylated vector were ligated overnight at 4°C with T4 ligase (New England Biolabs) prior to transformation in JM109 chemo-competent bacteria. Final plasmids were extracted and checked before sequencing.

Real-time PCR

After total mRNA extraction and purification with TRI REAGENT® (Sigma-Aldrich), mRNA were subjected to DNAse treatment (Ambion) with 0.25 µl DNAse per µg of RNA for 25 min at 25°C. Afterwards, 10 µg DNAse-treated mRNAs were purified in a phenol/chloroform/AIA solution (Fluka) complemented with 5% Sodium Acetate 3 M. 10% Sodium Acetate 3 M and 2.5 Volumes of 100% Ethanol were added to the aqueous phase and kept at -20°C overnight in order to precipitate mRNAs. After a brief wash in 70% Ethanol, pellets were left to dry and then re-suspended in 30 µl water. After an agarose gel check of mRNA quality, 2 µg of mRNA were subjected to reverse transcription as reported elsewhere [1]. Real-time quantitative PCR was performed on a Cfx C1000 system (Biorad). For each reaction, 12.5 ng of cDNA were placed in a final reaction mixture of 15 µl containing 7.5 µl of 2× SsoFast™ EvaGreen® Supermix (Biorad) and 200nM primer pairs (see Table S2). The housekeeping gene Glyceraldehyde-3-phosphate dehydrogenase (GAPDH) was used as an endogenous control to normalize variations in RNA extractions, the degree of RNA degradation and variability in RT efficiency. To quantify the results, we used the comparative Ct method. The PCR protocol was: an initial 30 sec denaturation step at 95°C, and 40 cycles of [4 sec at 95°C, 30 sec at 60°C] and a final dissociation curve to control the specificity of the amplification.

The respective quantification of full-length TRPM8 mRNA and 4TM-TRPM8 encoding mRNAs was performed as described: full-length TRPM8 mRNA was detected with primers targeting exons 7 and 8, whereas the sum of full-length TRPM8 and 4TM-TRPM8 mRNAs (in addition of TRPM8 mRNA) was measured with primers targeting exons 19 and 20. Note that, as the efficiency of the two primers' pairs was considered equal, the level of 4TM-TRPM8 mRNAs was estimated as the subtraction of ΔΔCt (7–8) value to ΔΔCt (19–20) value.

Immunoblotting

An ice-cold buffer (pH 7.2) containing 10 mM PO4Na2/K buffer, 150 mM NaCl, 1 g/100 ml sodium deoxycholate, 1% Triton X-100, 1% NP40, a mixture of protease inhibitors (Sigma-Aldrich), and a phosphatase inhibitor (sodium orthovanadate; Sigma-Aldrich) was applied to previously PBS-washed cells in dishes. After 30 min incubation on ice, the protein extract was transferred into 1.5 ml tubes and sonicated. After 10 minutes of centrifugation at 15,000 g, the pellet was transferred into a clean tube prior to a determination of the protein concentration using a BCA Protein Assay (Pierce). An SDS-page was performed using 25 µg of total protein loaded into a 10% polyacrylamide gel. After electrophoresis, proteins were transferred to a nitrocellulose membrane using a semi-dry electroblotter (Bio-Rad). The membrane was blocked in a TNT +5% (W/V) milk (15 mM Tris buffer, pH 8, 140 mM NaCl, 0.05% Tween 20, and 5% non-fat dried milk) for 30 min at room temperature, then soaked in the primary antibody diluted in TNT +1% milk for either 2 h at room temperature or overnight at +4°C. After three washes in TNT, the membrane was soaked in the secondary antibody diluted in TNT+1% milk for 1h at room temperature. The membrane was processed for chemiluminescence detection using Luminata Forte Western HRP Substrate (Millipore) according to the manufacturer's instructions. After a 10 min bath in Re-blot PLus Mild SOlution (Millipore), membrane was blotted again. The primary antibodies were: rabbit anti-TRPM8 (Ab109308, Abcam), rabbit anti-HA tag (Sc-805, Santa Cruz) and goat anti-GA3PDH (Sc-20357, Santa Cruz).

ImmunocytoFluorescence

Experiments were performed on LNCaP C4-2b cells plated on 35 mm glass bottom dishes (MatTek Inc) or on 6 µm-thick slices of the LNCaP C4-2b tumors obtained with Microm HM355S (Thermo Scientific Inc). After paraffin removal, antigen retrieval was achieved in a citrate buffer boiled 4 times for 5 minutes in a microwave oven and rinsed three times in PBS. Before immunocytofluorescence, LNCaP C-42b cells were fixed with 4% formalin in PBS for 10 min on ice prior to 3 PBS washes. Cells/tumors slices were subjected to blocking and permeabilization with PBS + 1.2% gelatine + 0.2% Tween + 0.2 M glycine for 30 min at 37°C. The slides/dishes were then incubated with primary antibodies 2 h at

37°C. After thorough rinsing in PBS/gelatine, the slides/dishes were treated with the corresponding secondary antibody: either Dye light 488-labeled anti-rabbit IgG (Jackson ImmunoResearch; dilution, 1/2000) or Alexa fluor 546-labeled anti-mouse IgG (Molecular Probes; dilution, 1/4000) diluted in PBS/gelatine for 1 h at ambient temperature. After rinsing twice in PBS/gelatine and once in PBS with 1/200 Dapi for 10 min at ambient temperature, the slides were mounted with Mowiol® and examined under a confocal microscope. The primary antibodies used were: rabbit anti-TRPM8 (Ab109308, Abcam), mouse anti-p21 (Clone SX118, Dako), rabbit anti- cytokeratin 14 (PRB-155P-100, Covance), rabbit anti- cytokeratin 5 (PRB-160P-100, Covance), mouse anti-cytokeratin 18 (Sc-51582, Santa Cruz) and mouse anti-Vimentin (Clone V9, Dako).

Apoptosis assay

Briefly, according to the manufacturer's protocols (TNR Red Roche), formalin-fixed and paraffin-embedded tissues tumor slices (6 μm) are incubated with PBS-G at 37°C in a humidified dark chamber. After TUNEL staining reaction (30 min, 37°C) in humidified dark chamber, slices are washed twice with PBS-G. Finally slices are staining with Hoechst/PBS (1/5000). Staining is visualized by LSM 700 confocal imaging system.

Flow cytometry

Flow cytometry was performed with a CyAn™ ADP Analyser. Cells were harvested, split into 1 million cell samples in 15 ml tubes before fixation with 1 ml of 70% Ethanol at –20°C overnight. Cells were washed twice with PBS/4% BSA 0,1% Triton ×100 and finally incubated at RT for 30 min. Cell were then pelleted by centrifugation at 250 × g for 8 min at 20°C.

For analysis of immunolabeled cell population, mouse anti- p21 (Clone SX118, Dako) primary antibody was diluted in 100 μl PBS-BT at 1/200 and incubated with cells at ambient temperature for 1 h. After a first quick wash in PBS, a second wash was done at RT for 30 min. Cells were incubated with rabbit anti-Ki67 antibody coupled to FITC (Ab27619, Abcam) and/or secondary antibodies specific to the host animal of the primary antibodies, antirabbit IgG coupled to Dye Light-488 (Jackson ImmunoResearch; dilution 1/2000) or anti-mouse IgG coupled to Alexa Fluor 647 (Jackson ImmunoResearch; dilution 1/4000) at RT for 30 min. After two PBS washouts for a total incubation time of 30 min at ambient temperature, cells were suspended in 500 μl of PBS and then analyzed.

For TUNEL (terminal deoxynucleotide transferasemediated dUTP—biotin nickend labelling) experiments, cells were pelleted and then suspended in 100 μl of labeling solution (TUNEL-TMR red, Roche) at room temperature for 30 min. Cells were washed once in PBS at ambient temperature for 10 min.

For cell cycle analysis, cells were suspended in 250 μl PBS/4% BSA/0,1% Triton X100 with Ribonuclease A (200 μg/ml) and incubated at room temperature for 15 min. After addition of 250 μl of PBS containing 30 μg/ml of propidium iodide, cells were further incubated at room temperature for 30 min to 1 hour. Data were analyzed with FlowJo software (version 8.7).

Apoptosis quantification

Alternatively to TUNEL experiments, cells were stained with 5 μg/ml Hoescht 33258 for 10 min at room temperature and mounted in glycergel (DAKO). Nuclear morphology (condensed and fragmented) was analyzed on an upright Axio Imager. A1 microscope (Zeiss, Germany). The percentage of apoptotic cells was determined by counting at least 500 cells in random fields.

Viability assay

Cells were transfected with siRNA in 100 mm dishes overnight. The day after, cells were dispatched in 96-well plates at a density of 5,000 cells per well in 100 μl RPMI medium. After 6 hours of incubation, experiments started as day 0, and treatments were applied by adding 100 μl of treatment-containing medium. From day 1, half of the medium was changed daily. CellTiter 96® AQ_{ueous} Non-Radioactive Cell Proliferation Assay (Promega) was used to determine the number of viable cells each day.

Wide-field Ca^{2+} imaging

Calcium imaging experiments have been performed as described previously [2]. Briefly, $[Ca^{2+}]_c$ was measured using ratiometric dye fura-2 (2 μM) and quantified according to the Grynkiewicz equation [3]. The bath solution (HBSS (Hank's Balanced Salt Solution)) contained 142 mM NaCl, 5.6 mM KCl, 1 mM $MgCl_2$, 2 mM $CaCl_2$, 0.34 mM Na_2HPO_4, 0.44 mM KH_2PO_4, 10 mM HEPES and 5.6 mM glucose. The osmolarity and pH of external solutions were adjusted to 310 $mOsm.l^{-1}$ and 7.4, respectively. The cells were continuously perfused with the HBSS solution and chemicals were added via a perfusion system.

Quantification of mitochondria Ca^{2+} content was carried out in HBSS (Hank's Balanced Salt Solution) containing 142 mM NaCl, 5.6 mM KCl, 1 mM $MgCl_2$, 2 mM $CaCl_2$, 0.34 mM Na_2HPO_4, 0.44 mM KH_2PO_4, 10 mM HEPES and 5.6 mM glucose. The osmolarity and pH of external solutions were adjusted to 310 $mOsm.l^{-1}$ and 7.4, respectively. Cytosolic Ca^{2+} concentration was measured using Fura2-loaded cells (2 μM) as described elsewhere [1]. The intracellular Ca^{2+} concentration was derived from the ratio of the fluorescence intensities for each of the excitation wavelengths (F340/F380) and from the Grynkiewicz et al. equation [3]. The cells were continuously perfused with the HBSS solution and chemicals were added *via* a perfusion system.

Confocal imaging

Confocal imaging was performed using a confocal microscope (LSM 780, Carl Zeiss Micro Imaging, Inc) with a plan-Apochromat 40 × /1.3 NA oil immersion objective and equipped with a thermo-controlled chamber. 405, 488, 561, 633 nm laser lines were used for excitation. Emission wavelengths were designed to produce no overlap between the four fluorophores. 1024*1024 pictures were acquired frame by frame with an averaging of 8 to reduce noise.

Time domain-fluorescence lifetime imaging microscopy (TM-FLIM)

For live-cell imaging, cells were placed on 35 mm glass bottom dishes (MatTek Corporation, USA), filled with L-15 medium without phenol red (Life technologies), and kept at 37°C using a stage incubator (Life Imaging Services, Switzerland). FLIM was performed with a Leica TCS SP5 X confocal head (Leica Microsystems, Germany) with the SMD upgrade, mounted on an inverted microscope (DMI6000, Leica Microsystems, Germany). A pulsed diode laser, PDL 800-B (PicoQuant GMBH, Germany), delivered 40 MHz repetitive rate pulses at 405 nm. The confocal pinhole was set to 1 Airy, for a 0.921 µm optical slice. Single photon events originated from the illuminated voxel were collected through a 63 × /1.2 NA water-immersion objective and recorded by a TCSPC detector (HydraHarp 400; PicoQuant GMBH, Germany). Fluorescence was detected through a 483/32 single-bandpass filter (Semrock, USA) on Single Photon Avalanche Photodiodes, SPAD (MPD, Italy), set up at 256 × 256 pixels. Arrival time of single photons was measured with SymPhoTime software (PicoQuant GMBH, Germany) while image were taken with LAS AF software (Leica Microsystems, Germany). In order to obtain the best resolution of organelles, a 5-fold zoom factor was applied, giving a pixel size of 0.193 µm and an image size of 49.21 × 49.21 µm. Since the statistical determination of the distribution of single photon arrival time requires a minimum of 100 photons per pixel, 120 frames were acquired at 200 Hz and summed in the final image. Fluorescence lifetime of the donor of fluorescence was determined by the Phasor plot method using a homemade software [4].

Since fluorescence phase lifetime is independent of the concentration of the fluorescence emitter, the FRET-FLIM measurement of a biosensor limits artifacts due to the variation of concentration in a single cell or between different cells. FRET efficiency value, namely E (FRET), was figured out from eq(1)

$$\text{Eq(1)} \quad E_{(\text{FRET})} = 1 - \frac{\tau_{DA}}{\tau_D}$$

and directly correlates to the proportion of Ca^{2+}-bound Cameleon, then it consequently correlates to steady-state [Ca^{2+}].

SupplementaryTable S1: List of siRNA (sense sequence) used in the study

siRNA label	5′-3′ RNA
siCTL	CUUACGCUGAGUACUUCGA
siM8-4b	CAAGAAACACACCAAGGAA
siM8-6a	GGAGUUCAGAGGAGAAUAU
siM8-6a(M1)	GGAGUUCAGAAGAGAAUAU
siM8-6a(M3)	GGAGUUCAGAGAAGAAUAU
siM8-6a.2	GGGGAGGUGGUGAGAGAUA
siM8-7	UCUCUGAGCGCACUAUUCA
siM8-10	GGGAUGAAAUUGUGAGCAA
siM8-12	GGAAACUGGUUGCGAACUU
siM8-19	GUAUUCUGGACGAGUCAUU
siM8-20	UAUUCCGUUCGGUCAUCUA
siPERK (B2, Darhmacon)	UAGCAAAUCUUCUUCUGAA
siPERK (D2, Darhmacon)	ACUAAUCGAUUGCAUAUUG

SupplementaryTable S2: List of qPCR primers and cloning primers used in the study

qPCR primers	5′-3′ forward	5′-3′ backward
Trpm8(Ex7–8)	CTGTCATGGACATCCCACTG	GGGATCTTGCCACCATAGTT
Trpm8(Ex19–20)	ATTCCGTTCGGTCATCTACG	CACACACAGTGGCTTGGACT
Gapdh	ACCCACTCCTCCACCTTTG	CTCTTGTGCTCTTGCTGGG
Atf4	AGTGCCTGCGGCAGCATTGG	CAACGCTGCTGCTGAATGCCG
Atf6	TGAAGCCATCCGCAGAAGGGGA	GGGTGGTAGCTGGTAACAGCAGG
Hspa5	AGAAACCGCTGAGGCTTATTTGGGA	TTGGTTGCTTGGCGTTGGGC
Ddit3	AAAGATGAGCGGGTGGCAGCG	AGCTGCCATCTCTGCAGTTGGAT
Eif2ak3	GCTGTCGGACCTCGCAGTGG	TCCGGCTCTCGTTTCCATGTCTG
Hsp60	TGGTGCAGATGCCCGAGCCTT	GGGACTTCCCCAACTCTGCTCAA
Xbp1	ACAGCGCTTGGGGATGGATGC	GGGGTGACAACTGGGCCTGC
Cdkn1a	TCAGGGTCGAAAACGGCGGC	TTTGAGGCCCTCGCGCTTCC
Cdkn1b	AGCGGAGCAATGCGCAGGAA	GGCGTCTGCTCCACAGAACCG
Pcna	GACACCTACCGCTGCGACCG	AGCGCCAAGGTATCCGCGTT
Dnaj	AGAACGCTCGGTGAGAGGCGG	CGGTGTGTGAGGGAGCGGGAA
Txnrd2	GGTGGTCGGCGGGGGATCTG	TTCCACGTAGTCCACCACGGC
Hsp10	AGTCGCTGTTGGATCGGGTTCT	TGGTGCCTCCATATTCTGGGAGA

Cloning	5′-3′ forward	5′-3′ forward
Trpm8	GAGAGACCAGCAGGATCCTTGG	TCAAGGTCTCAGCACACTAGG
Trpm8 (15a)	GTGCTGATGTCGCTGTAGAGC	TCAAGGTCTCAGCACACTAGG
Trpm8 (16a)	GAAGAAAGTTTGCATGGCATCCTG	TCAAGGTCTCAGCACACTAGG
Trpm8 (17′)	GTGTGCATTTAGCTACTAAGTCAC	TCAAGGTCTCAGCACACTAGG
Trpm8 (17″)	AGGGACATGGGGTGGGAGT	TCAAGGTCTCAGCACACTAGG
Trpm8 (18a)	GGACATTTAAAAATCTGGAAATGGTTG	TCAAGGTCTCAGCACACTAGG
sM8γ	GAGAGACCAGCAGGATCCTTGG	GAGATTGCTGAGAACACATTTTAATGAAC
sM8α	TGACCTGTGGGAAGTGGCACTG	GAGATTGCTGAGAACACATTTTAATGAAC
sM8ε	ATGGAGAGAGAAGAGGAACATCAG	GAGATTGCTGAGAACACATTTTAATGAAC
sM8η	GAAGTTGGGAGGGAATGCTAAAC	GAGATTGCTGAGAACACATTTTAATGAAC

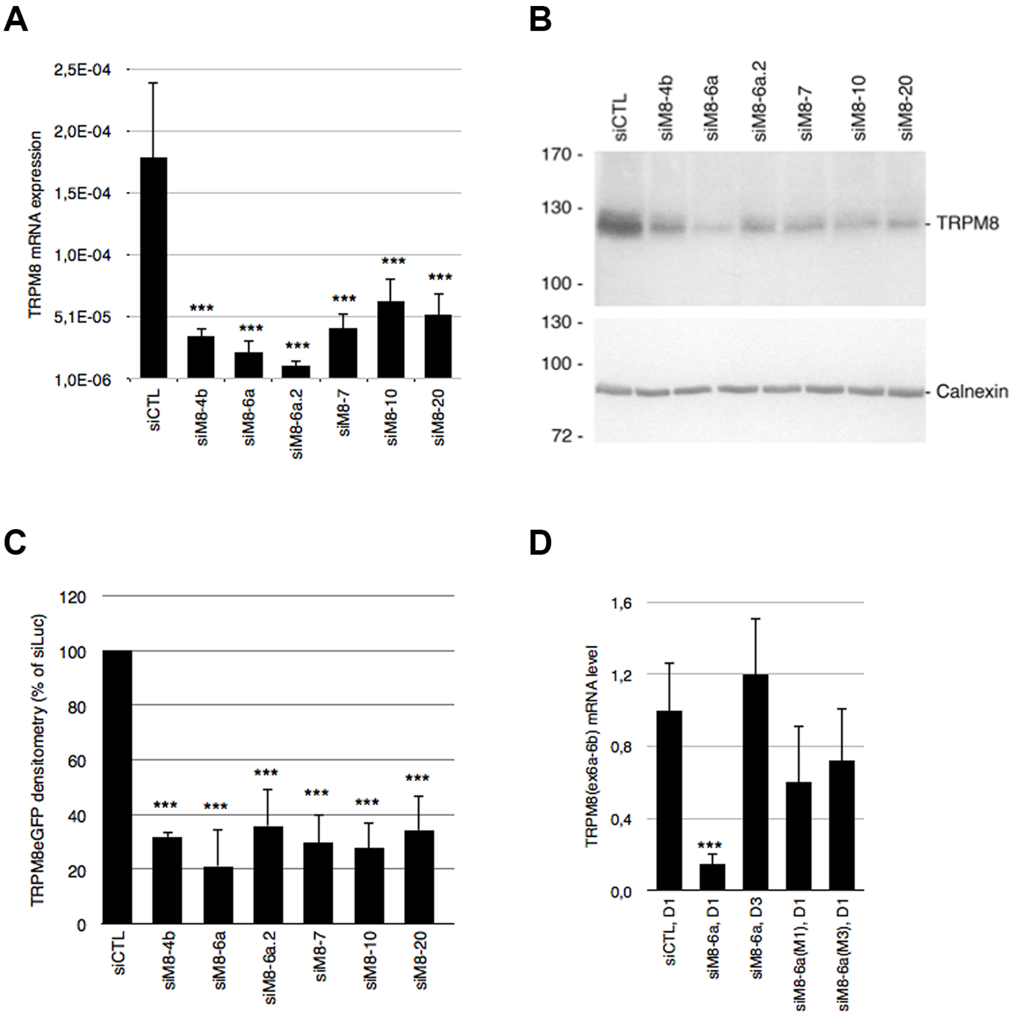

Supplementary Figure S1: Control of silencing efficiency of anti-TRPM8 siRNAs. (**A**) Bar diagram plot shows the quantification of TRPM8 mRNA level in full-length TRPM8 inducible HEK cells after a 2-days transfection with anti-TRPM8 siRNAs. (**B**) Western-blot shows expression of full-length TRPM8 channel in TRPM8 inducible HEK cells after a 3-days transfection of anti-TRPM8 siRNAs. 50 µg of total protein extract were loaded on each well. (**C**) Bar diagram plot represents the quantification of TRPM8 proteins detected by immunoblotting by densitometry. Values are normalized on siLuc value. (**D**) Silencing of native short TRPM8 (sM8) mRNAs in LNCaP C4-2b cells transfected with siRNA for 2 days. TRPM8 mRNA level has been measured from exon 6a to exon 6b to specifically detect sM8 transcripts, and were quantified by the mean of qPCR. Values are normalized on siLuc value. All experiments were performed three times independently. Values are expressed as Mean ± SD.

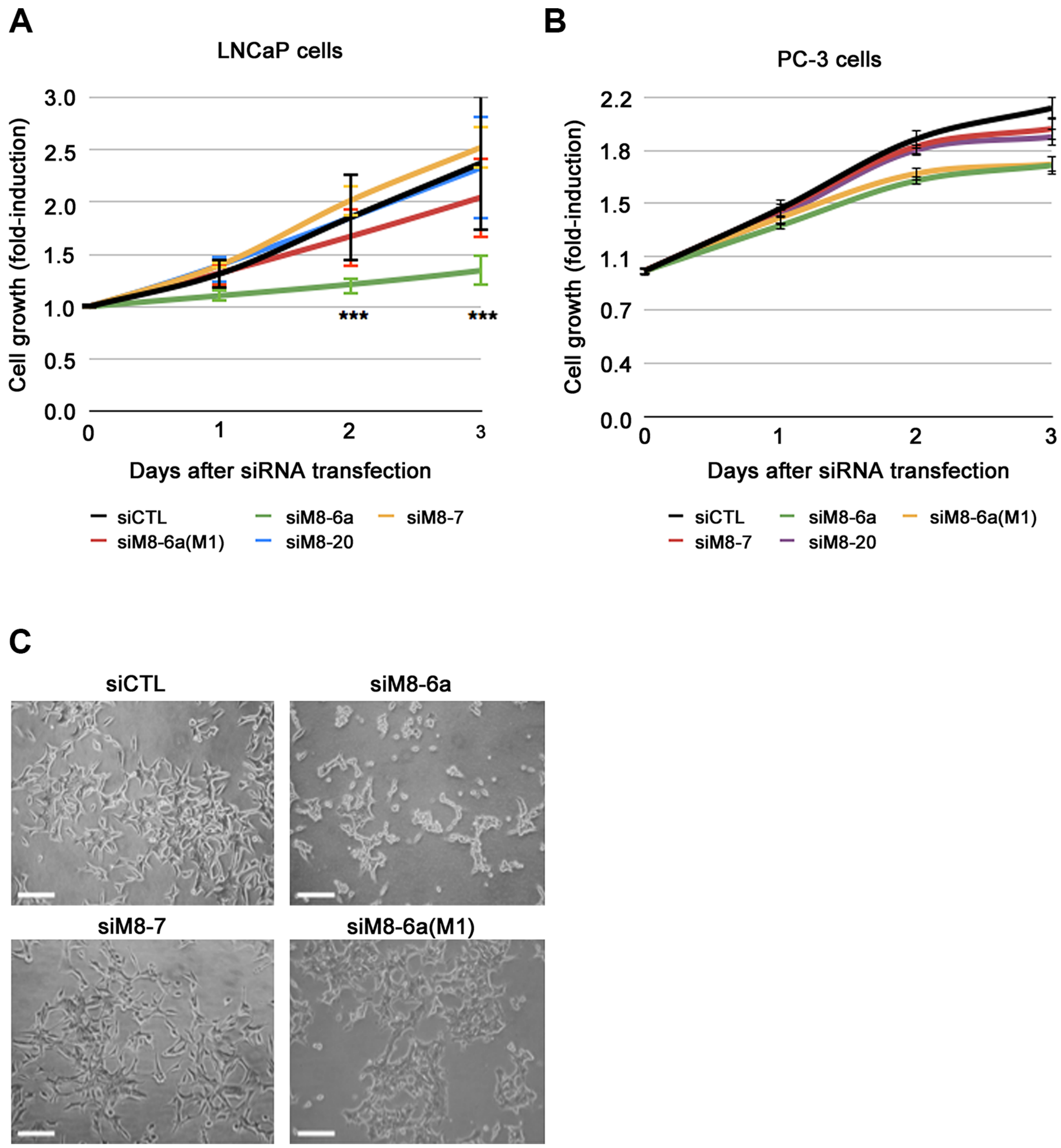

Supplementary Figure S2: Suppression of sM8 isoforms inhibits cell growth of androgen-dependent LNCaP and androgen-refractory LNCaP C4-2b cells. (**A**) and (**B**) Growth kinetic of LNCaP and PC-3 cell lines, respectively. siRNA targeting selective groups of TRPM8 isoforms were transfect at day 0. Estimation of cell number was achieved usin the CellTiter 96® AQ_{ueous} Non-Radioactive Cell Proliferation Assay (Promega), as described in Materiels and Methods. Experiments were performed three times independently. Values are expressed as Mean ± SD. (**C**) Phase-contrast images of LNCaP C4-2b cells after a 3-day siRNA transfection. Scale bar represents 50 µM.

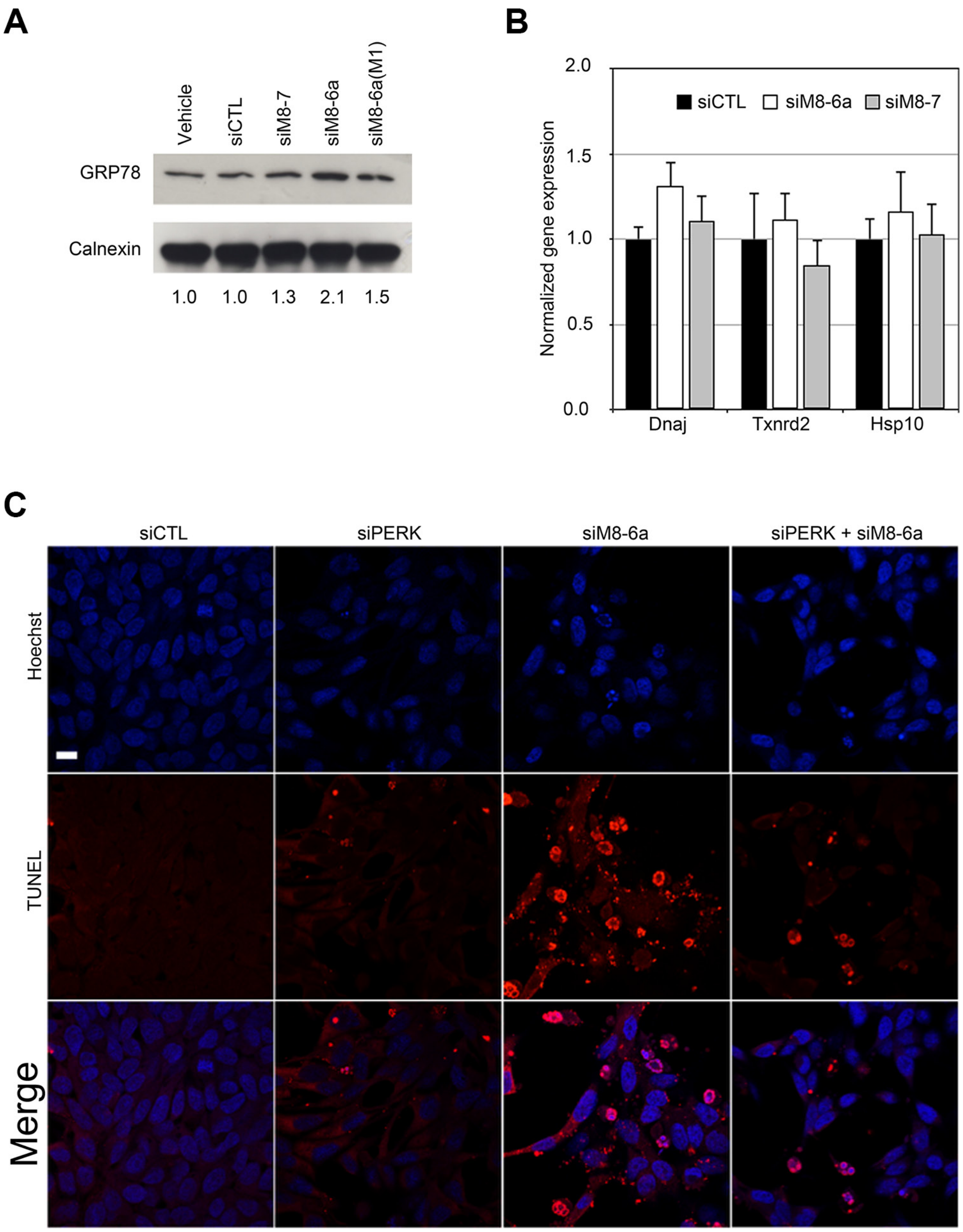

Supplementary Figure S3: sM8 knockdown does not modify expression of genes implicated in mitochondrial stress. (**A**) Representative western-blot reports induction of ER-stress marker, GRP78, in LNCaP C4-2b cells transfected with siM8-6a specifically. Calnexin protein was used as a protein-loading reporter. Values indicate GRP78 levels normalized on Calnexin levels. (**B**) Expression of genes involved in mitochondrial stress: Dnaj, Txrnd2, Hsp10 was estimated with qPCR after a 3-day siRNA transfection of LNCaP C4-2b cells. Experiments were performed three times independently. Values are expressed as Mean ± SD. (**C**) The concomitant suppression of PERK protein (siPERK) and sM8 proteins (siM8-6a) decrease sM8 KD-induced apoptosis in LNCaP C4-2b cells. All cells were stained with Hoechst (top row) and apoptotic cells were labeled with TUNEL-TMR (middle row). Merged images are shown in the down row. Scale bar: 5 µm.

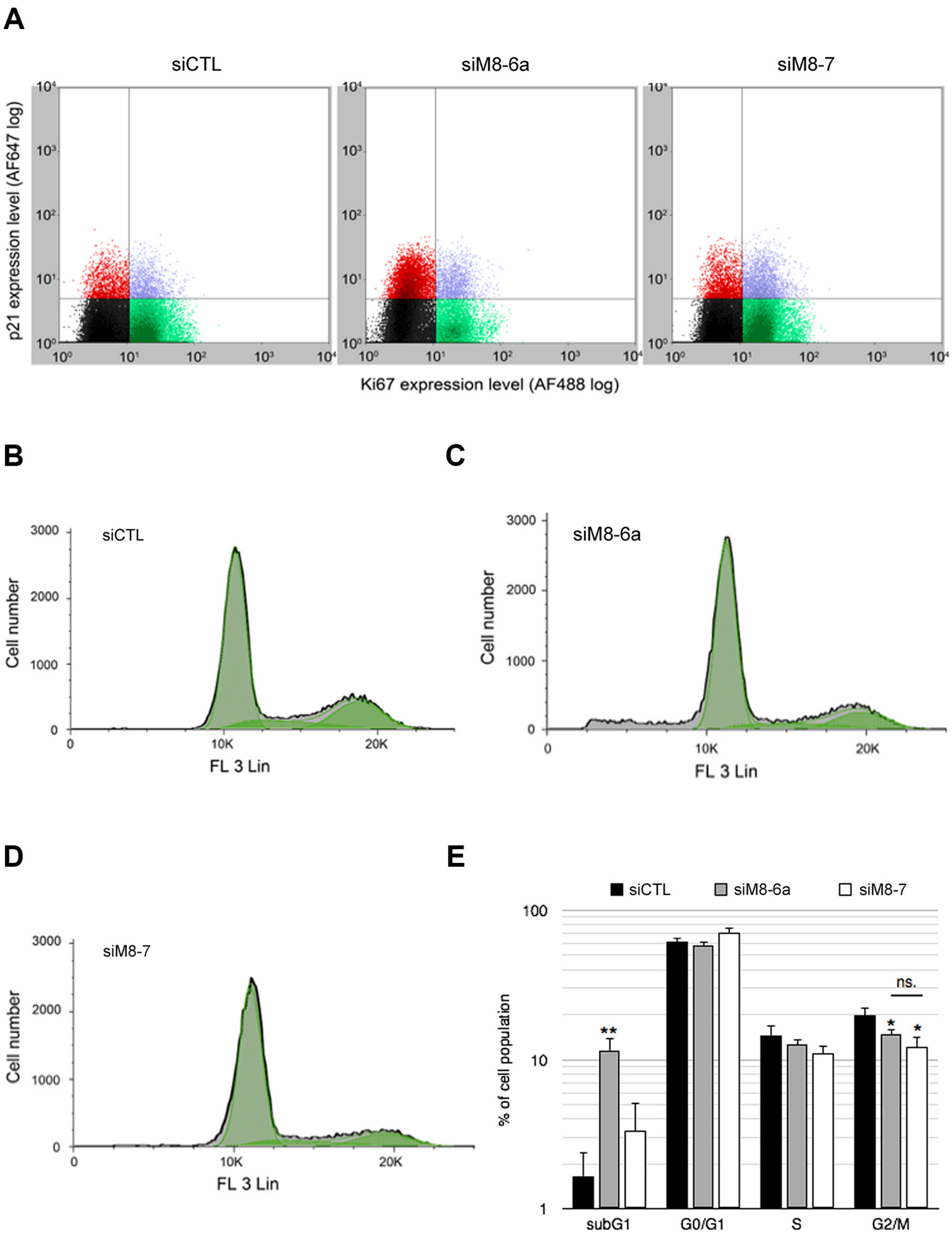

Supplementary Figure S4: sM8 knockdown does not significantly modify cell cycle distribution of LNCaP C4-2b cells. (**A**) shows the distribution of p21 and Ki67 immunolabelled cells reported by flow cytometry (representative experiment shown). Red area represents the p21+/Ki67- cell population, while the violet area represents the p21+/Ki67+ cell population and the green area p21-/Ki67+ cell population. Cell cycle analysis by flow cytometry was performed in LNCaP C4-2b cells transfected with siLuc (**B**), siM8-6a (**C**) or siM8-7 (**D**) for 3 days. Distributions of cells by their DNA content (FL 3 lin, x axe) are fitted with the Dean-Jett-Fox model to estimate the cell sub-populations in subG1, G0/G1, S and G2/M phases. Experiments were performed three times and average values of the cell sub-populations were figured out. (**E**) Bar diagram plot represents the mean ± SD of the cell sub-population at the different phase of the cell cycle.

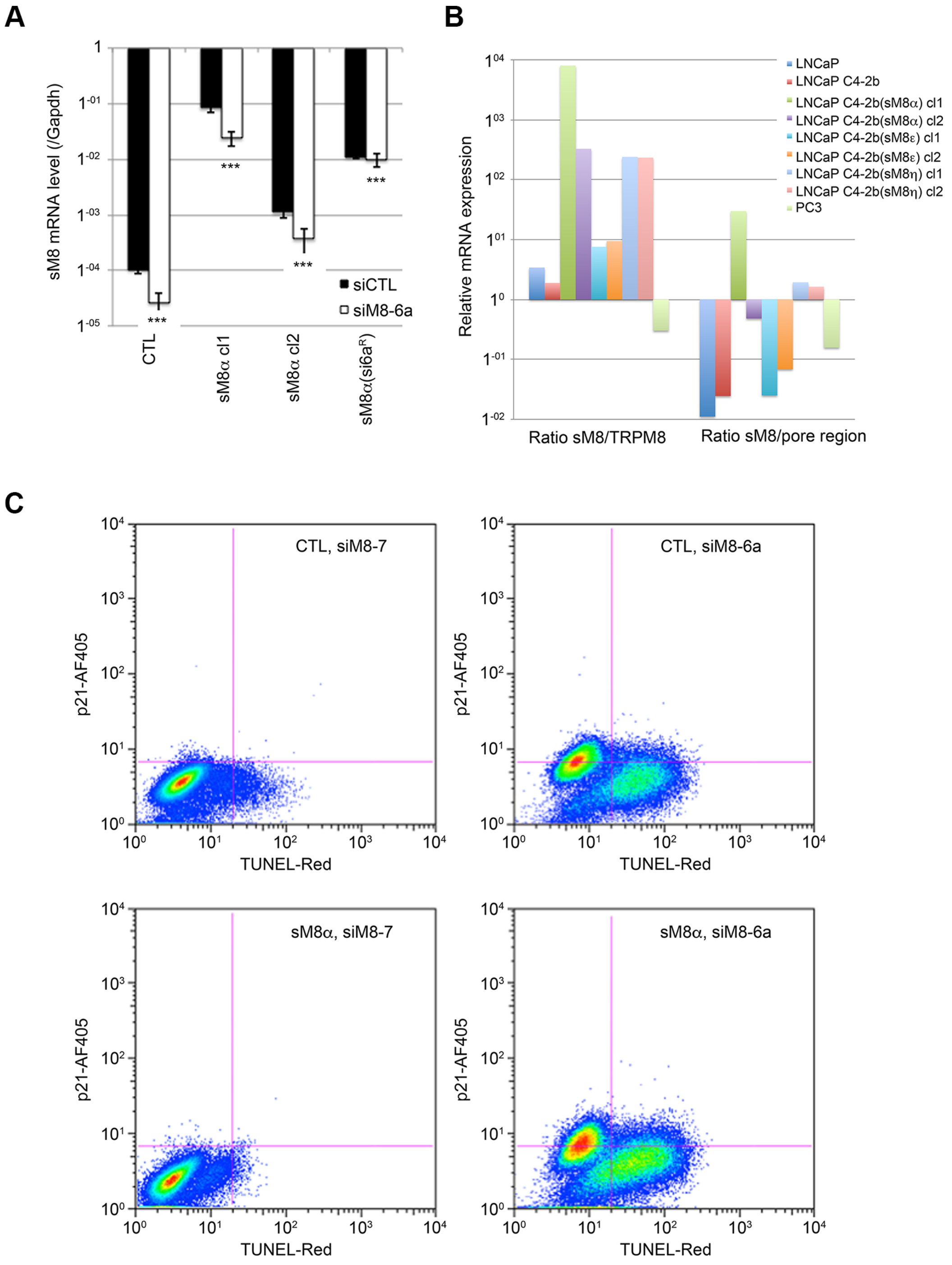

Supplementary Figure S5: (**A**) Quantification of sM8 mRNA levels in control LNCaP C4-2b clone 1 and in LNCaP C4-2b clones 1 and 2 overexpressing sM8α, or in LNCaP C4-2b clone overexpressing the mutant sM8α insensitive to sM8-6a-induced sM8 knockdown, sM8α (si6a^R). Cells were transfected 2 days with siM8-6a siRNA before qPCR analysis was performed. (**B**) Relative expression of sM8 mRNA on either TRPM8 mRNA or on detection of the pore region (including TRPM8 and 4TM-TRPM8) in different cell lines. Ratios were calculated by dividing the expression of sM8, TRPM8 and pore region normalized on Gapdh level. (**C**) Distribution of p21-immunolabeled and TUNEL-positive cells reported by flow cytometry (representative experiment shown) in a population of LNCaP C4-2b cells (CTL) or sM8 α-overexpressing C4-2b clone 1 transfected with either siM8-7 or siM8-6a. Pseudo-color code represents cell density from the lowest (blue) to highest (red). Experiments were performed three times independently.

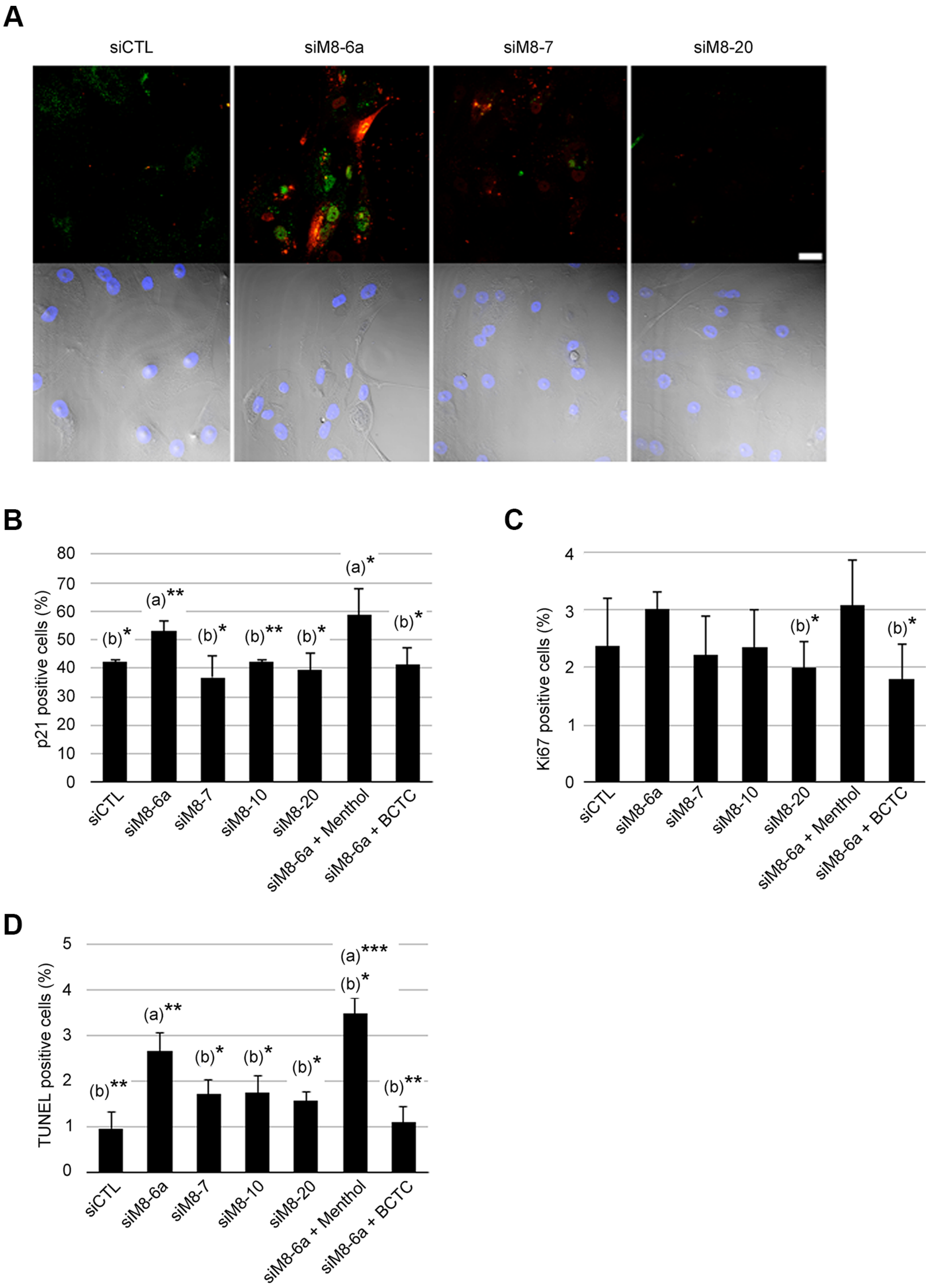

Supplementary Figure S6: sM8 knockdown results in p21 induction and increase of apoptosis in primary cultures of prostate epithelial cancer cells. (**A**) Immunocytofluorescence, on the top, shows the concomitant TUNEL labeling of apoptotic nuclei (red) and immunodetection of p21 (green) in a primary culture of prostate epithelial cancer cells. Cells were treated with TRPM8 mRNAs-targeting siRNAs in addition of menthol or BCTC, an agonist and an antagonist, respectively, of TRPM8 and 4TM-TRPM8 channels. Below are presented merged images of bright field and dapi fluorescence (405 nm). Panels (**B**), (**C**) and (**D**) represent the percentage of p21 positive cells, the percentage of Ki67 positive cells and the percentage of TUNEL positive cells, respectively, after transfection with siRNA (for details see Materials and Methods). Menthol (500 µM) or BCTC (10 µM) have been added daily in the medium of cells transfected with siM8-6a for the 3 last days of the experiment. Experiments were performed three times independently. Values are expressed as Mean ± SD.

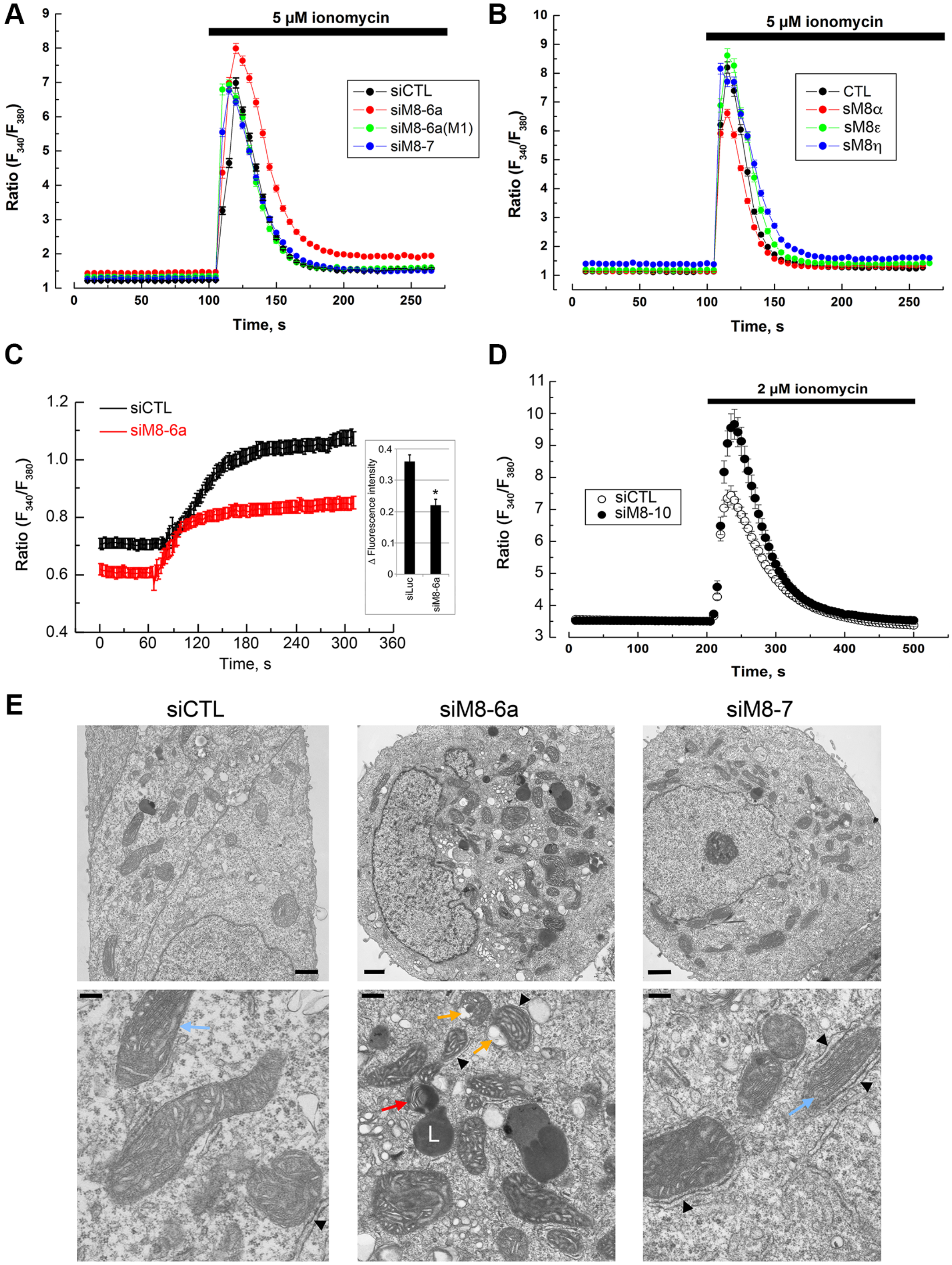

Supplementary Figure S7: Modification in ER and mitochondria calcium content after suppression or overexpression of sM8. (**A**) Time course of Ca^{2+} imaging experiments realized on LNCaP C4-2b cells 3 days after transfection with 50 nM of siRNAs targeting luciferase (si Luc, *n* = 387) or TRPM8 (siM8-6a, *n* = 348; siM8-6a(M1), *n* = 184 and siM8-7, *n* = 345). Experiments were carried out in the absence of extracellular calcium. (**B**) Time course of Ca^{2+} imaging experiments carried out in different LNCaP C4-2b clones: control clone (CTL), *n* = 344; sM8α, *n* = 307; sM8ε, *n* = 302; sM8η, *n* = 280. (**C**) Quantification of mitochondria Ca^{2+} content based on FCCP-induced liberation in LNCaP C4-2B cells after a 2-day transfection with siM8-6a-mediated silencing of sM8s isoforms compared to a control siRNA (siCTL). FCCP (5 µM) was applied to Ca^{2+}-free. Therefore, the transient $[Ca^{2+}]$c rise reflects the amounts of calcium liberated from the total mitochondria compartment. Mean ± Sem, *n* > 100 for each condition. (**D**) Same as A. in LNCaP C4-2b cells transfected with siM8-10 (*n* = 177) vs siCTL (*n* = 223). (**E**) Top row: TEM micrographs of LNCaP C4-2b cells after a 2-day transfection with siCTL, siM8-6a or siM8-7. Scale bar: 2µm. Bottom row: higher magnification TEM micrographs. Blue arrow: normal mitochondria in LNCaP cells. Swollen mitochondria (orange arrow) or a mitochondria undergoing mitophagy (red arrow in close contact with a lysosome (L) are visible in sM8s KD LNCaP cells (siM8-6a). Black arrowheads show the mitochondria associated membranes of ER. Scale bar: 0.5 µm. Experiment was reproduced three times.

REFERENCES

1. Gackiere F, et al. A role for voltage gated T-type calcium channels in mediating “capacitative” calcium entry? Cell Calcium. 2006; 39:357–66.
2. Thebault S, et al. Novel role of cold/menthol-sensitive transient receptor potential melastatine family member 8 (TRPM8) in the activation of store-operated channels in LNCaP human prostate cancer epithelial cells. J Biol Chem. 2005; 280:39423–35.
3. Grynkiewicz G, Poenie M, Tsien RY, A new generation of Ca2+ indicators with greatly improved fluorescence properties. J Biol Chem. 1985; 260:3440–50.
4. Leray A, et al. Quantitative comparison of polar approach versus fitting method in time domain FLIM image analysis. Cytometry A. 2011; 79:149–58.
